# (Thio)urea-mediated synthesis of functionalized six-membered rings with multiple chiral centers

**DOI:** 10.3762/bjoc.12.48

**Published:** 2016-03-10

**Authors:** Giorgos Koutoulogenis, Nikolaos Kaplaneris, Christoforos G Kokotos

**Affiliations:** 1 Department of Chemistry, National and Kapodestrian University of Athens, Panepistimiopolis 15771, Athens, Greece

**Keywords:** multiple chiral centers, organocatalysis, six-membered ring, thiourea, urea

## Abstract

Organocatalysis, now running its second decade of life, is being considered one of the main tools a synthetic chemist has to perform asymmetric catalysis. In this review the synthesis of six-membered rings, that contain multiple chiral centers, either by a ring closing process or by a functionalization reaction on an already existing six-membered ring, utilizing bifunctional (thio)ureas will be summarized. Initially, the use of primary amine-thioureas as organocatalysts for the above transformation is being discussed, followed by the examples employing secondary amine-thioureas. Finally, the use of tertiary amine-thioureas and miscellaneous examples are presented.

## Introduction

**Scheme 1:**
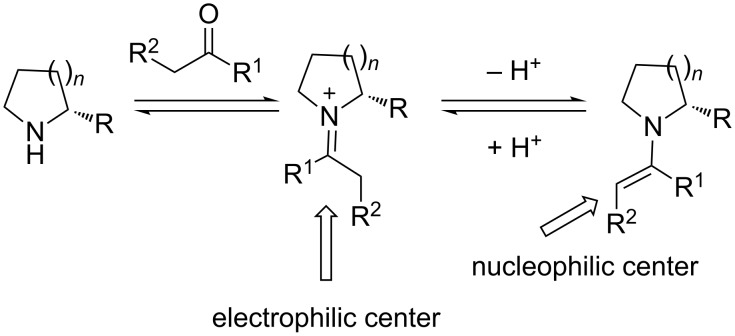
Activation of carbonyl compounds via enamine and iminium intermediates [2].

During the last 15 years, organocatalysis has flourished and has been established as one of the three major pillars of asymmetric synthesis [1–3]. Among the modes of activation of organic molecules that have been designed and developed, the functionalization of carbonyl compounds via enamine and iminium ion intermediates are the most common [4–5] (Scheme 1).

The carbonyl compound condenses with the amino catalyst, to form an iminium ion, subsequent deprotonation leads to the highly nucleophilic enamine. This kind of intermediates have been proposed to be the reactive intermediates in many reactions such as aldol, Michael, Mannich, and α-functionalization (α-chlorination, α-amination, α-fluorination) reactions. Proline-type organocatalysts are considered priviliged, because their corresponding enamines exist mainly in the *s-trans* conformation, that factor is crucial since complete prediction of the stereochemical outcome of the reaction is possible.

**Scheme 2:**
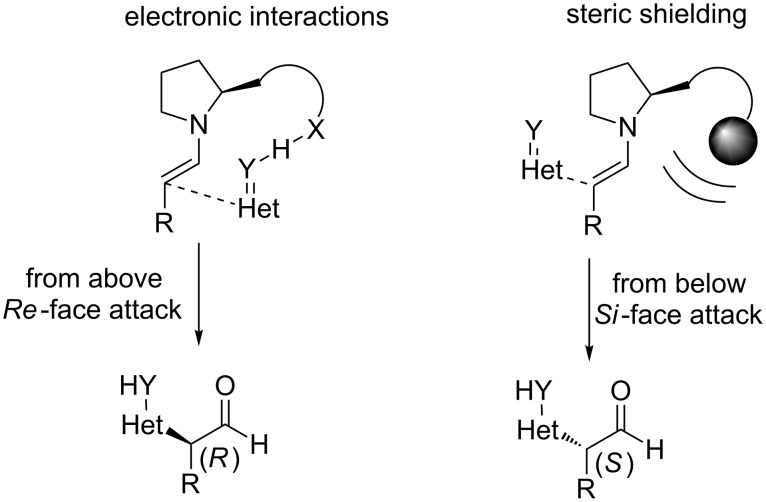
Electronic and steric interactions present in enamine activation mode [2].

Generally, the enamines formed can interact with the substrates in two ways, via electronic or steric interactions (Scheme 2). The electronic interaction depicted in the left, in Scheme 2, seems to be operative, when the R group of the organocatalyst possesses a moiety, that is able to form hydrogen bonds, being the hydrogen bond donor. Employing this logic, many organocatalysts have been developed, possessing various groups, that are able to form hydrogen bonds, such as carboxylic acids, tetrazoles, thioureas, etc. The selectivity observed, when steric shielding interaction is employed, is due to the bulky group of the catalyst. This group shields one face of the enamine to provide the selectivity.

**Scheme 3:**
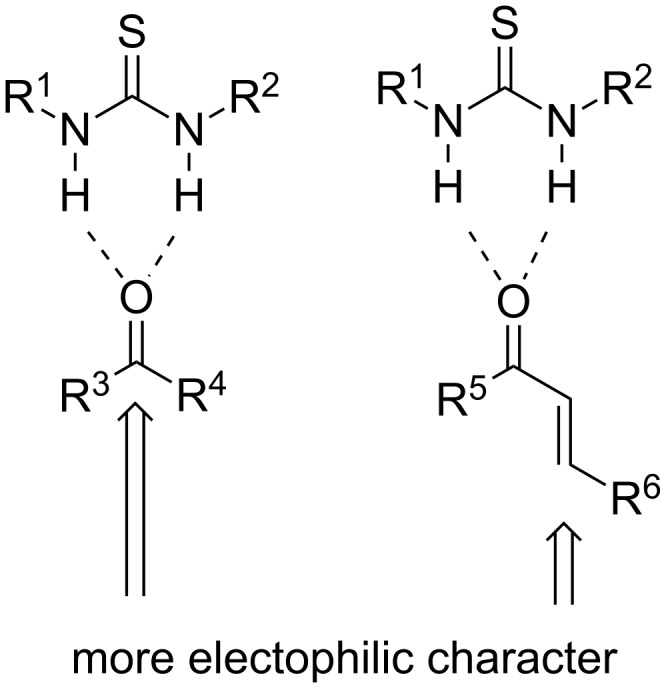
Electrophilic activation of carbonyl compounds by a thiourea moiety.

The third most valuable and studied mode of activation involves hydrogen bonding, which is also postulated to be present in enzymatic reactions. (Thio)urea moieties have been employed in order to activate electrophiles and in order to allign them, in a specific manner, so as to react with nucleophiles (Scheme 3) [6–7]. In addition, many bifunctional (thio)ureas have been synthesized in order to utilize both hydrogen bonding interactions and enamine formation. In the last 10 years the field has witnessed the development of some new activation modes, such as SOMO catalysis [8] and photoredox organocatalysis [9].

Six-membered rings are found in many natural products, pharmaceuticals and agrochemicals, thus, a lot of effort has been put by the synthetic community to provide mild, reliable, robust and operationally simple methods to construct them. Of the vast variety of six-membered rings, those with multiple chiral centers pose the most difficult synthetic challenge, because not only the regiochemical outcome, but also the stereochemical outcome of the reaction must be carefully controlled. Since its rebirth, organocatalysis has made many contributions in the synthesis of six-membered rings with multiple chiral centers, this area has been reviewed in the past [10–14]. This review will focus on (thio)urea organocatalysts, including primary, secondary and tertiary amine groups. Miscellaneous catalysts will be also presented. Thus, it will provide an exhaustive overview of this area, rather than providing a few examples of each class of organocatalysts.

## Review

### Primary amine-thioureas as organocatalysts promoting asymmetric transformations that lead to a six-membered ring

As discussed earlier, except from the activation of the substrates with the formation of the corresponding enamines or iminium ions, the synthesis of enantiopure products can be also achieved organocatalytically with hydrogen bonding. Organocatalysts that contribute to hydrogen bond formation bear usually a urea or thiourea moiety and they mostly interact with carbonyl groups, nitro moieties or even imines that exist to the substrates, leading to increased electrophilicity; urea and thiourea moieties have also been proposed to interact with nucleophiles. Besides the fact that hydrogen bond donors increase the electrophilicity of the substrates, they mostly coordinate the transition state of the reaction, controlling this way the stereoselectivity of the products. It has been postulated that as the acidity of component HX is increased, the stronger the resulting hydrogen-bonding interaction Y···H–X is [15]. As a logical conclusion, it seems that multiple hydrogen-bonding interactions will provide a more defined conformation to the transition state, thus the catalysts, which contain urea or thiourea moieties are more efficient. If someone combines the ability of amines, to form the corresponding enamines with a carbonyl compound and the ability of ureas or thioureas to define a specific conformation in the transition state of the reaction, then, one can take advantage of a bifunctional catalyst. The first family of these bifunctional catalysts, that are going to be discussed, are the "primary amine-thioureas".

**Scheme 4:**
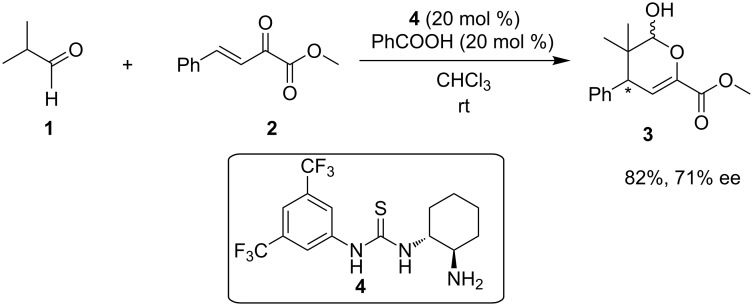
Asymmetric synthesis of dihydro-*2H*-pyran-6-carboxylate **3** using organocatalyst **4** [16].

Initially, catalyst **4** was studied as an organocatalyst in the addition of isobutyraldehyde (**1**) to *(E)*-methyl 2-oxo-4-phenylbut-3-enoate (**2**) for the formation of substituted dihydro-*2H*-pyran-6-carboxylate **3** (Scheme 4) [16]. It was observed, that by employing PhCOOH as an additive, the yield (%) and the ee (%) increased, in comparison to the use of 4-dimethylaminopyridine (DMAP). A single example was shown leading to 82% yield and an enantiomeric excess of 71%. The suggested mechanism for this catalytic reaction involves a bifunctional activation.

**Scheme 5:**
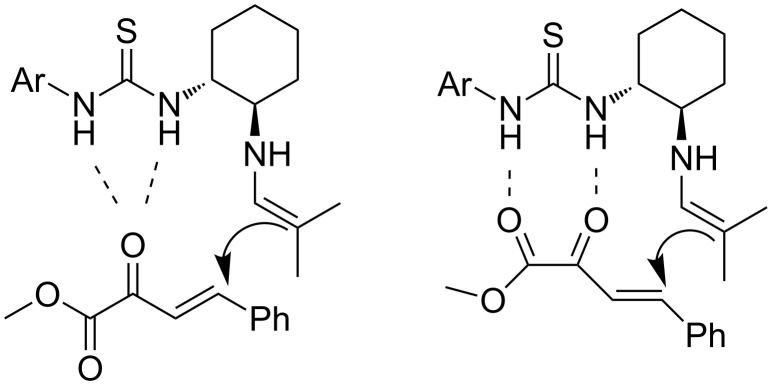
Possible hydrogen-bonding for the reaction of *(E)*-methyl 2-oxo-4-phenylbut-3-enoate [16].

Utilizing the primary amino group, the authors proposed that the catalyst condenses to form an imine, which is in equilibrium with the corresponding enamine of isobutyraldehyde, while the two hydrogens of the thiourea group interact with one or two carbonyl groups of phenylbutenoate **2** (Scheme 5).

**Scheme 6:**
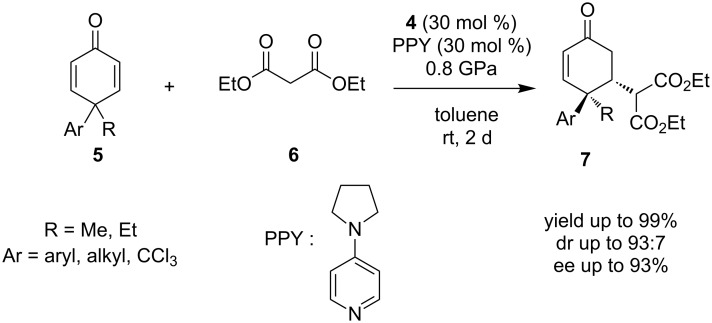
Asymmetric desymmetrization of 4,4-cyclohexadienones using the Michael addition reaction with malonates [17].

Another catalytic reaction catalyzed by a primary amine-thiourea that leads to multiple chiral centers is the asymmetric desymmetrization of 4,4-disubstituted cyclohexadienones **5**, using the Michael addition of malonates **6**, to obtain 3,4,4-trisubstituted cyclohexanones **7** [17]. It is noted that the organocatalyst employed is the same with the previous example, catalyst **4**. Furthermore, this reaction is taking place in the presence of PPY and high pressure was utilized. The authors proposed that PPY deprotonates the ethyl malonate, producing the active nucleophile, while the thiourea group activates the electrophile (Scheme 6). The above catalytic reaction provided products with yields up to 99%, dr up to 93:7 and ee up to 93%.

**Scheme 7:**
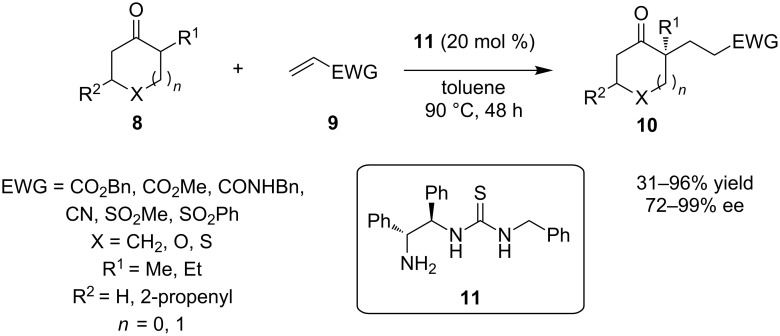
The enantioselective synthesis of α,α-disubstituted cycloalkanones using catalyst **11** [18].

Carter and co-worker utilized a similar primary amine-thiourea, organocatalyst **11**, in an enantioselective synthesis of α,α-disubstituted cycloalkanones **10**. Starting from α-substituted cycloalkanones **8** and alkenes **9**, containing an electron withdrawing group, α,α-disubstituted cycloalkanones were obtained (Scheme 7) [18]. The reaction described above provided products with yields up to 96%, ee up to 98% and complete regiocontrol. The authors proposed that the primary amino group of the organocatalyst condenses with the ketone, to form the corresponding enamine, which in turn reacts with the electrophilic alkene **9**.

**Scheme 8:**
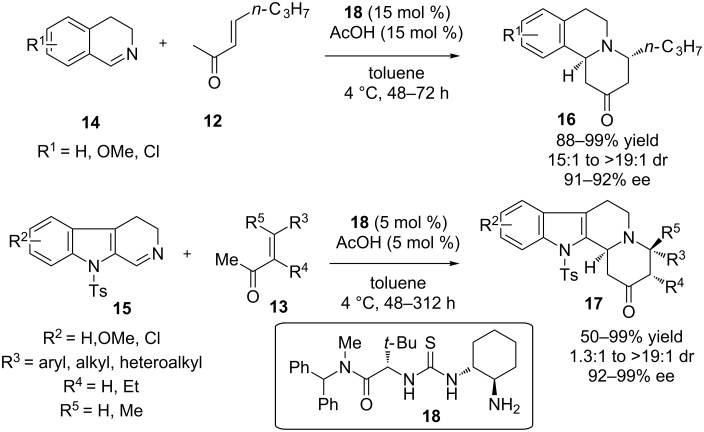
The enantioselective synthesis of indolo- and benzoquinolidine compounds through aza-Diels–Alder reaction of enones with cyclic imines [19].

Jacobsen and co-workers have introduced a number of (thio)ureas as organocatalysts for a variety of transformations. Utilizing the primary amine-thiourea **18**, an enantioselective formal aza-Diels–Alder reaction of enones **12** and **13** was reported. In this reaction the enamine is formed from the side of the methyl ketone, which is conjugated with the pre-existing double bond, providing the electron-rich diene, which reacts with substituted dihydroisoquinoline **14** and dihydro-β-carboline **15**, so that cyclohexanone derivatives **16** and **17** will be produced, respectively (Scheme 8) [19]. Also, a cyclic derivative of **13** was utilized (not shown). This aza-Diels–Alder reaction provides products with yields up to 99% and up to 99% ee. This constitutes an excellent addition in a synthetic chemist’s arsenal for the synthesis of polycyclic heterocycles.

**Scheme 9:**
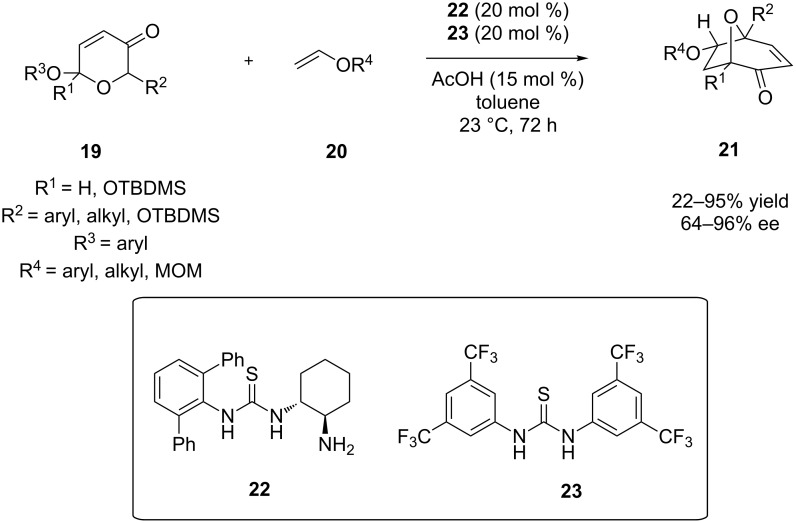
Enantioselective [5 + 2] cycloaddition [20].

Along the same lines of cycloadditions, Jacobsen and co-workers reported the combination of a primary amine-thiourea **22** and an achiral thiourea catalyst, organocatalyst **23**. More specifically, the reaction is a catalytic asymmetric synthesis of 8-oxabicyclooctanes via an intermolecular [5 + 2] pyrylium cycloaddition (Scheme 9) [20]. This novel [5 + 2] cycloaddition describes the coupling of a pyrylium ylide **19** with dipolarophile **20**, in order to give access to the 8-oxabicyclo[3.2.1]octane **21** framework. In this reaction, the main factor of achieving high yields or enantioselectivities, is how electron-rich or electron-poor, the dienophile is. Electron-rich olefins, like the benzyl vinyl ether and ethyl vinyl ether, reacted with success providing high yields and high enantiomeric excess. It has been observed that a nucleophilic 2π-reactant is needed for the successful conversion of the reactants into the desired products, following a mechanism which involves a cationic, electron-poor amino-pyrylium intermediate. In addition, for the achievement of high ee values the nature of R^3^ is very important. The better the leaving group R^3^ is, the higher the values of the ee. It is mentioned that the ee in this reaction is up to 96%, and the recommended R^3^ group to be used is 3,4,5-trifluorobenzyl.

**Scheme 10:**
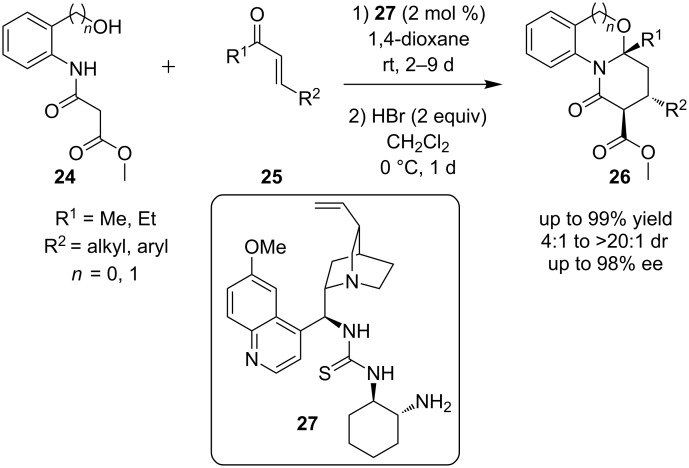
Asymmetric synthesis of oxazine derivatives **26** [21].

The use of the bifunctional amine-thiourea catalyst **27**, into a reaction providing oxazine derivatives **26**, was reported by Ye and co-workers (Scheme 10) [21]. In this reaction, nucleophile **24** is coupled to arylenone **25** to give the desired product. Initially a Michael reaction is taking place, followed by cyclization. After screening of various acids, hydrobromic acid was found to be the optimum acid for the second step of the reaction. Products were obtained in good to excellent yields (64–99%), with >20:1 diastereoselectivity and excellent values of up to 98% ee.

**Scheme 11:**
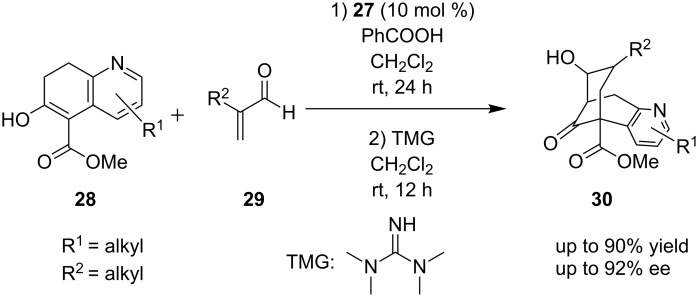
Asymmetric synthesis of bicyclo[3.3.1]nonadienone, core **30** present in (−)-huperzine [22].

Employing the same catalyst as before, organocatalyst **27**, another synthesis of the bridged core **30** and specifically the bicyclo[3.3.1]nonadienone of (−)-huperzine was reported (Scheme 11) [22]. The reagents were the analogue of pyridine **28** and an α,β-unsaturated aldehyde **29**. In order to obtain the desired products, an α-substituted α,β-unsaturated aldehyde must be used. In this particular reaction, the product was obtained in 78–90% yield and 15–92% ee. Finally, β-substituted α,β-unsaturated aldehydes were completely unreactive.

**Scheme 12:**
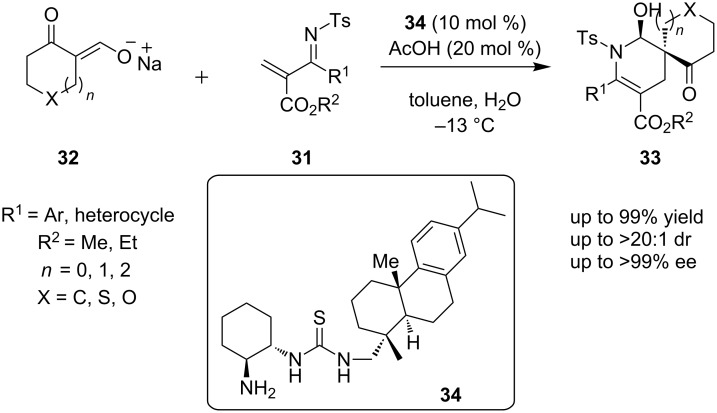
Asymmetric inverse electron-demand Diels-Alder reaction catalyzed by amine-thiourea **34** [23].

In 2012, a proposed inverse electron-demand Diels–Alder reaction was reported by Wang and co-workers, obtaining enantiopure products **33**, starting from diene **31** and dienophile **32**, using compound **34** as the catalyst (Scheme 12) [23]. This reaction provided products in 84–99% yield and with a diastereoselectivity of up to >20:1 and excellent enantioselectivity (88–99% ee).

**Scheme 13:**
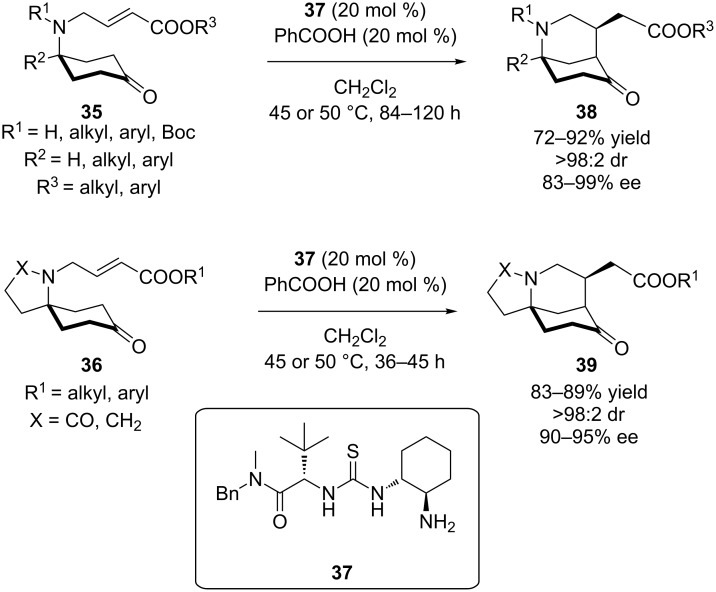
Asymmetric entry to morphan skeletons, catalyzed by amine-thiourea **37** [24].

In 2015, Dixon, Paton and co-workers demonstrated an elegant route to morphan skeletons, utilizing prochiral ketones **35** or **36**, catalyzed by a primary amine-thiourea **37** developed by Jacobsen. The proposed pathway is based on desymmetrization of **35** or **36** by an intramolecular Michael addition of the corresponding enamines to an α,β-unsaturated ester, to yield bicyclic or spiro-bicyclic products **38** and **39**, respectively, in excellent yields and stereoselectivities (Scheme 13) [24]. Computational studies were employed, in order to support the mechanistic pathway and the origins of stereocontrol.

### Secondary amino-thioureas as organocatalysts promoting asymmetric transformations that lead to a six-membered ring

**Scheme 14:**
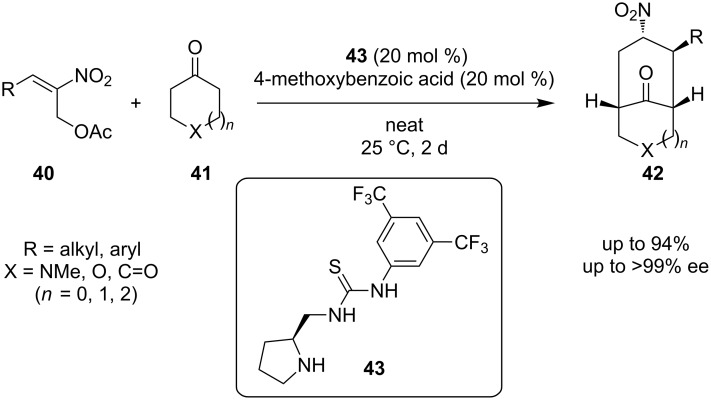
Asymmetric transformation of *(E)*-2-nitroallyl acetate [25].

**Scheme 15:**
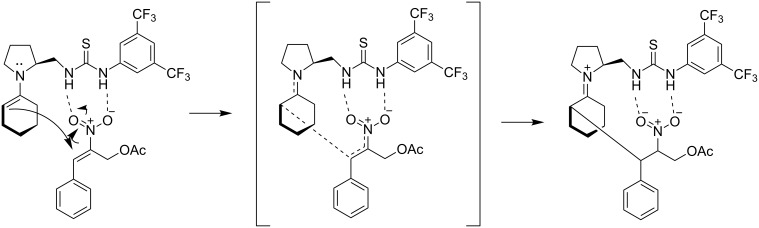
Proposed way of activation.

In 2009, the first asymmetric tandem reaction for the construction of bicyclic skeletons utilizing a secondary amine-thiourea was reported (Scheme 14) [25]. In this reaction, *(E)*-2-nitroallyl acetates **40** were used, that could serve as reagents, which can install a nitro group into the final product. After screening of various catalysts, organocatalyst **43** and 4-methoxybenzoic acid as a cocatalyst, was identified as the optimum for the reaction of *(E)*-2-nitroallyl acetate **40** with cyclohexanone **41** to provide nitrobicyclo[3.3.1]nonan-9-one **42**, in solvent-free conditions. This reaction provides products with yields up to 94% and enantiomeric excess up to >99%. A proposed mechanism for this reaction is shown below, where the formation of the *s-trans*-enamine occurs and then attacks the electrophilic double bond of the nitroallyl acetate (Scheme 15).

**Scheme 16:**
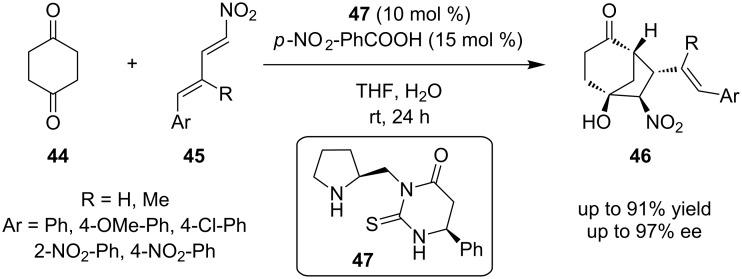
Asymmetric synthesis of nitrobicyclo[3.2.1]octan-2-one derivatives [26].

Among the same lines, Tsakos and Kokotos reported an enantioselective domino-Michael–Henry reaction catalyzed by a secondary amine-thiourea between cyclohexa-1,4-dienone (**44**) and a γ,δ-alkyl-aryl-disubstituted nitrodiene **45**, providing bicyclo[3.2.1]octan-2-one **46** (Scheme 16) [26]. The organocatalyst used in this reaction is the cyclic thiourea **47**. It is noted, that organocatalyst **47** affords products only in organic solvents and more specifically in THF. This tandem Michael–Henry reaction provided the product in an excellent yield of 91%, excellent enantiomeric excess of 96% and complete diastereocontrol.

**Scheme 17:**
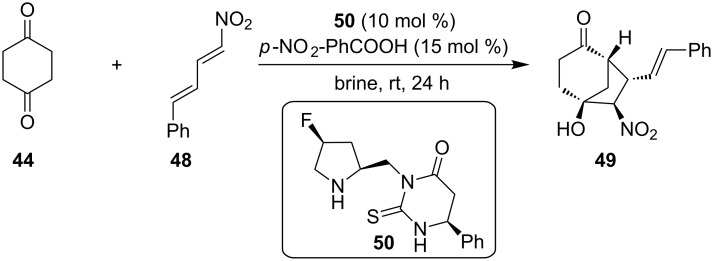
Asymmetric tandem Michael–Henry reaction catalyzed by **50** [27].

Trying to provide a greener alternative, Kokotos and co-workers, catalyzed the same tandem Michael–Henry reaction between cyclohexa-1,4-dienone (**44**) and nitrodiene **48** by employing the secondary amine-thiourea **50**, which contains a fluorine on its skeleton and 4-nitrobenzoic acid as a cocatalyst, to provide the substituted bicyclo[3.2.1]octan-2-one **49** (Scheme 17) [27]. It is highly noted that the difference to the moiety at the 4-position of the pyrrolidine ring, where a fluorine atom exists, gives to the organocatalyst **50** the ability to catalyze this tandem Michael–Henry reaction in brine, giving excellent diastereoselectivity and enantiomeric excess, unlike the previously employed catalyst **47**, which worked only in organic solvent. The key component for the achievement of catalyst’s **50** catalytic ability is the known "gauche effect" of fluorine in the pyrrolidine ring, where σ*(C–F) and σ(C–H) vicinal orbitals tend to overlap [28]. For a more efficient overlap of these two orbitals the ring has a certain bent conformation, which presumably makes the formed enamine more planar and a better nucleophile to attack the nitrodiene. This tandem Michael–Henry reaction provided the product in a medium yield 48%, excellent enantiomeric excess 97% and excellent diastereoselectivity >99:1.

### Tertiary amine-(thio)ureas as organocatalysts promoting asymmetric transformations that lead to a six-membered ring

#### One-step reactions producing six-membered rings

**Scheme 18:**
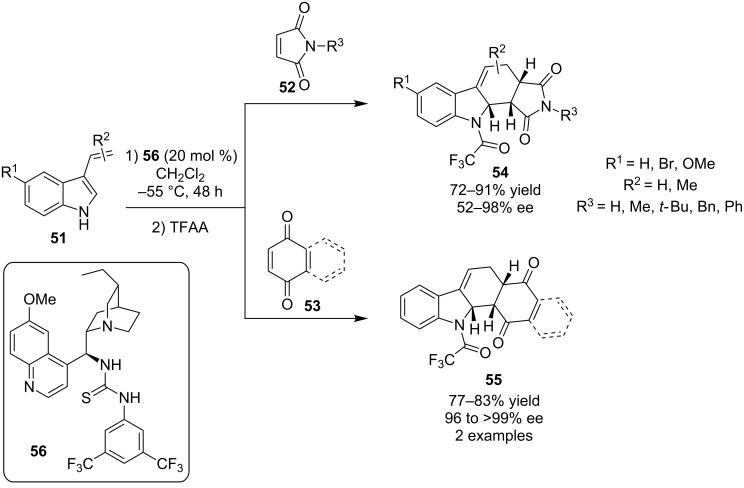
Asymmetric Diels–Alder reactions of 3-vinylindoles **51** [29].

In 2008, the first example of a single reaction producing a six-membered ring with multiple stereocenters catalyzed by a tertiary amine-thiourea **56** was reported by Bernardi, Ricci and co-workers for the Diels–Alder reaction of 3-vinylindoles **51** (Scheme 18) [29]. The authors utilized either maleimides **52** or quinones **53** as the dienophile, affording the products **54** and **55** in excellent yields and enantioselectivities, after trapping of the adducts with trifluoroacetic anhydride (TFAA), in order to make the products more stable. As expected the *endo*-adduct was the sole product observed.

**Scheme 19:**
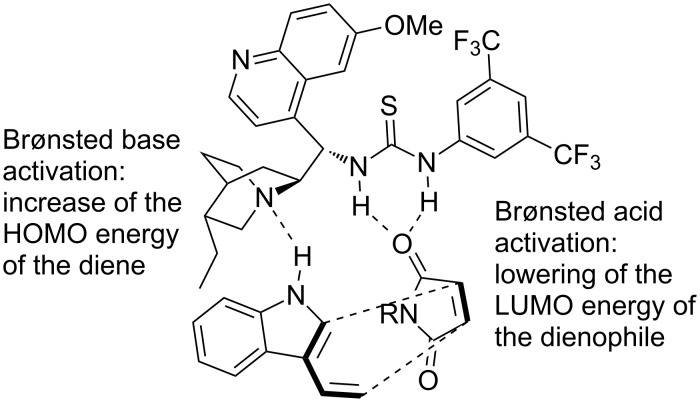
Proposed transition state and activation mode of the asymmetric Diels–Alder reactions of 3-vinylindoles **51** [29].

For this transformation, quinine-derived bifunctional organocatalyst **56** was utilized. The authors proposed that the catalyst raises the HOMO of the nucleophile, making the diene more nucleophilic, and lowers the LUMO of the electrophile, making the dienophile more electrophilic (Scheme 19), thus the catalyst acts via a bifunctional mode. All these interactions are developed in the transition state through hydrogen-bonding, which controls the stereochemical outcome of the reaction.

**Scheme 20:**
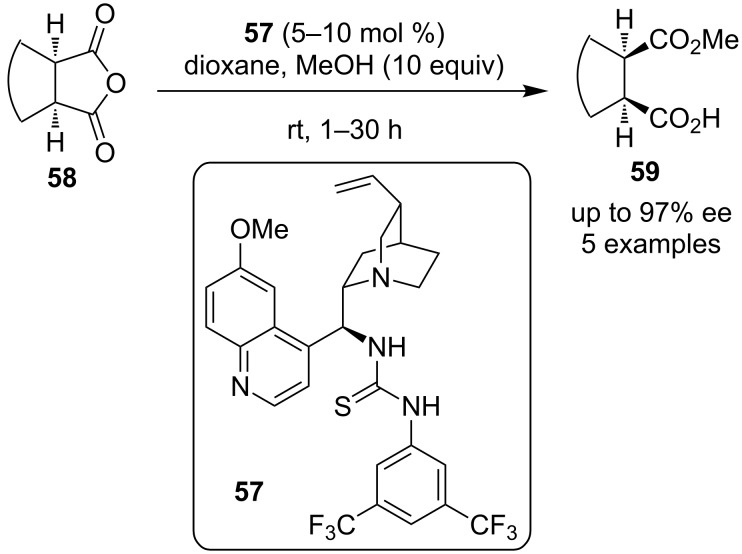
Desymmetrization of *meso*-anhydrides by Chin, Song and co-workers [30].

**Scheme 21:**
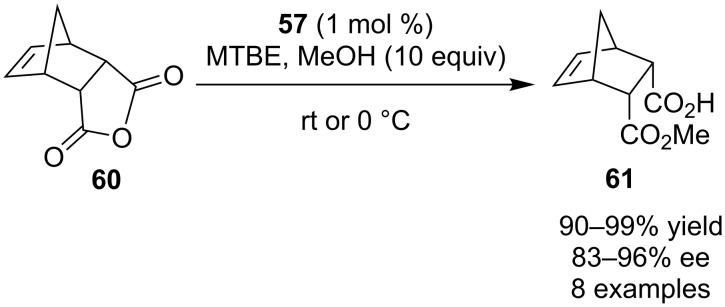
Desymmetrization of *meso*-anhydrides by Connon and co-workers [31].

The same year, two different groups utilized thiourea catalyst **57** to catalyze the desymmetrization of *meso* anhydrides **58** and **59** through a methanolysis reaction (Scheme 20 and Scheme 21).

Chin, Song and co-workers utilized the catalyst in 5–10 mol % catalyst loading and dioxane as solvent, producing the desired products **59** in excellent enantioselectivities [30].

Connon and co-workers, on the other hand, changed slightly the catalytic system, using only 1 mol % catalyst loading and MTBE as solvent to afford products **61** in excellent yields (90–99%) and good to excellent enantioselectivities (83–96% ee) [31].

**Scheme 22:**
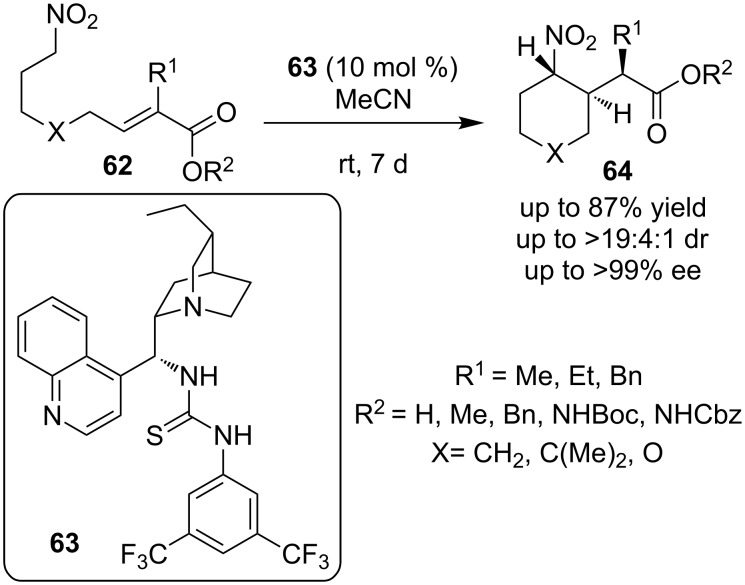
Asymmetric intramolecular Michael reaction [32].

In 2009, Cobb and co-workers disclosed the asymmetric intramolecular Michael addition of nitronates **62** onto conjugated esters utilizing the cinchona-derived thiourea **63** (Scheme 22) [32]. The reaction proceeded with excellent selectivity and afforded products **64** in good yield. The substrate scope of this reaction was thoroughly studied and the products of the transformation were exploited to generate a variety of γ-amino acids, including examples containing three contiguous stereocenters.

**Scheme 23:**
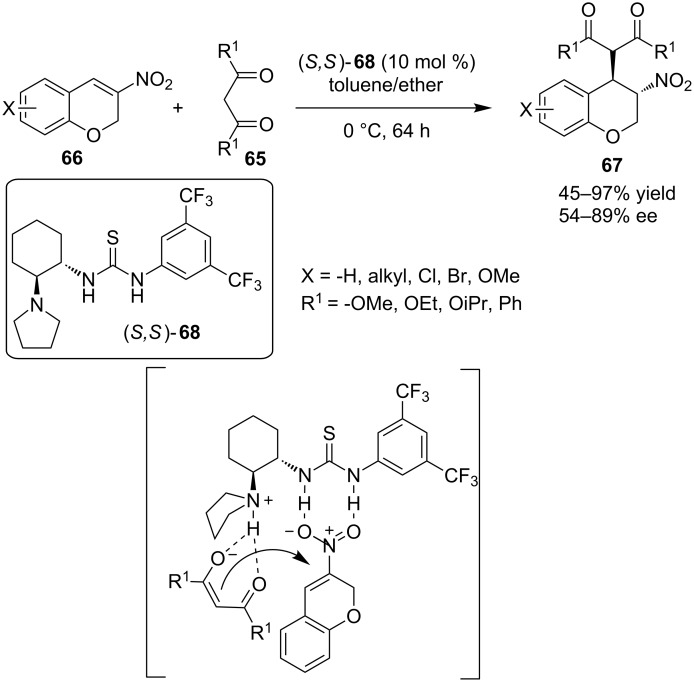
Asymmetric addition of malonate to 3-nitro-2*H*-chromenes **67** [33].

In 2010, Yan and co-workers described the Michael addition of malonates **65** to 3-nitro-2*H*-chromenes **66**, which provided the substituted chromanes **67** in moderate to excellent yields and good enantioselectivities (Scheme 23) [33]. Catalyst (*S,S*)-**68** is postulated to catalyze the reaction in a bifunctional manner: the tertiary amine deprotonates the malonate and the resulting enolate is directed to the upper face of the 3-nitro-2*H*-chromene due to hydrogen bonding of the enolate with the ammonium cation. The thiourea moiety, firstly activates the 3-nitro-2*H*-chromene through two hydrogen bonds, making it more electrophilic (LUMO lowering effect) and secondly it orients it near the enolate.

**Scheme 24:**
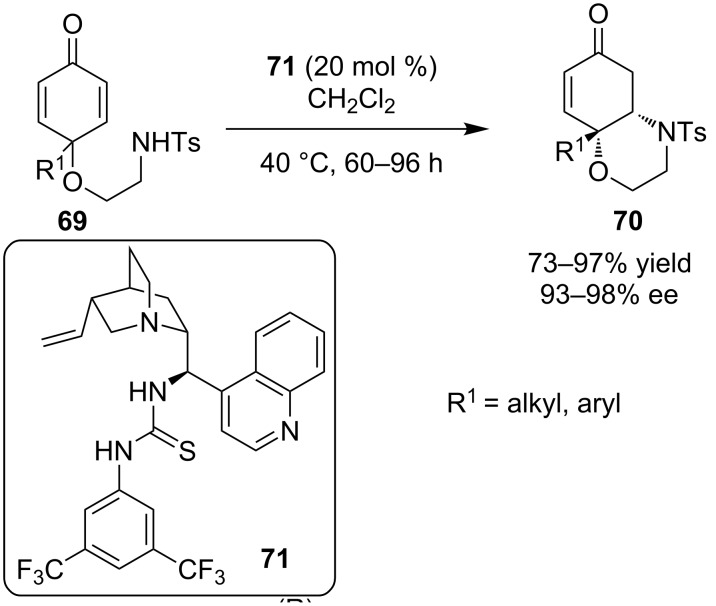
Intramolecular desymmetrization through an intramolecular aza-Michael reaction [34].

In 2011, You and co-workers described the intramolecular desymmetrization of cyclohexadienones **69** catalyzed by thiourea **71**, derived from cinchonine to give a bicyclic system **70** containing two chiral centers, utilizing an aza-Michael reaction (Scheme 24) [34]. The reaction proceeded in good to excellent yield and excellent enantioselectivity for almost all of the substrates that were tested.

**Scheme 25:**
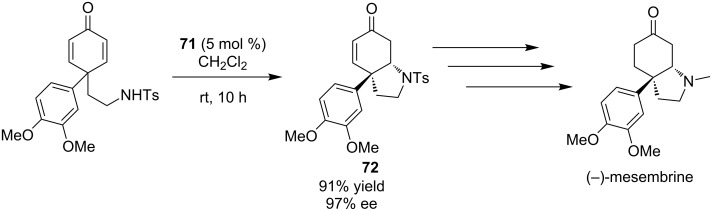
Enantioselective synthesis of (−)-mesembrine [34].

This methodology was further extended in the total synthesis of (−)-mesembrine. This natural product contains a sterically hindered and arylated quaternary carbon center, which was constructed via a desymmetrization aza-Michael reaction. That key intermediate **72** was afforded in 91% yield and 97% ee. (Scheme 25).

**Scheme 26:**
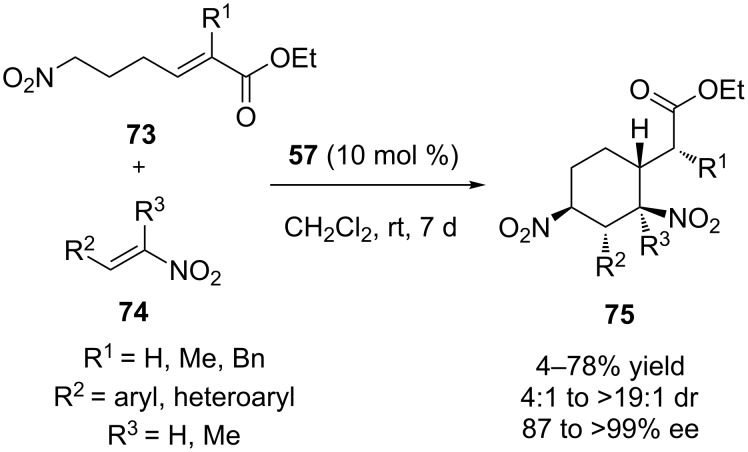
A novel asymmetric Michael–Michael reaction [35].

In 2012, Cobb and co-workers developed a novel asymmetric Michael–Michael reaction between nitrohex-4-enoates **73** and nitroolefins **74** to construct a cyclohexene moiety, bearing multiple contiguous stereocenters, including one quaternary center [35]. The reaction proceeded smoothly and a wide range of products **75** were obtained in good yields and moderate to excellent stereoselectivity (Scheme 26). The authors proposed that the organocatalyst deprotonates substrate **73** to produce a nitronate, which reacts with the electrophilic nitroolefin via a Michael addition. The resulting nitro compound is again deprotonated by the organocatalyst and reacts with the α,β-unsaturated ester to yield the desired product.

### Cascade/domino/tandem reactions producing six-membered rings

Cascade and tandem reactions always seemed very appealing to the synthetic community, not only because of their elegance, but also for their efficiency [36–42]. Cascade and tandem reactions have been proven extremely efficient because in only one synthetic operation, many bond-forming steps are achieved. Organocatalysis has made many contributions in cascade and tandem processes [43–45], due to the mild conditions required for the organocatalysts to operate, many distinct reactions can be conducted in one-pot fashion.

#### Cascade/domino/tandem reactions producing six-membered rings initiated by Michael addition

**Scheme 27:**
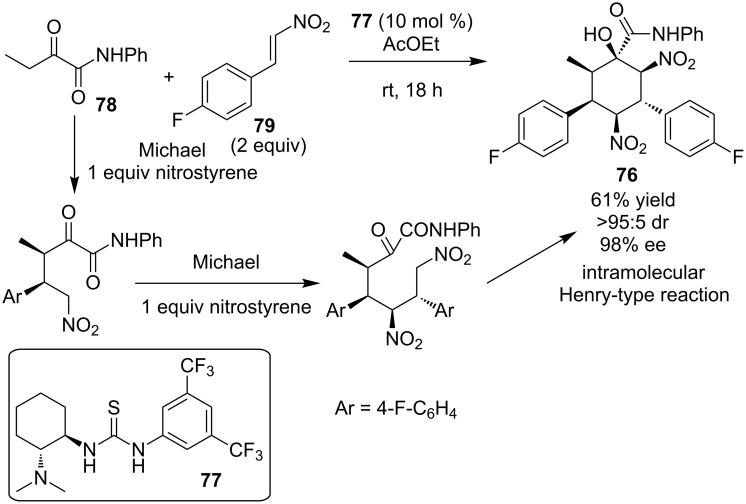
Asymmetric three-component reaction catalyzed by Takemoto’s catalyst **77** [46].

Bonne, Constantieux, Rodriguez and co-workers reported an enantioselective three-component Michael–Michael–Henry reaction to access a highly substituted cyclohexane **76** with excellent selectivity over three steps (>95:5 dr, 98% ee) using Takemoto’s catalyst **77** (Scheme 27) [46]. The cascade starts with a Michael addition of the enol of the α-keto-amide **78** to nitroalkene **79**, subsequent Michael addition of nitronates to the second equivalent of nitroalkene **79** and finally a Henry-type reaction between nitronate and the highly electrophilic carbonyl of the α-keto-amide, resulting in the final product **76**.

**Scheme 28:**
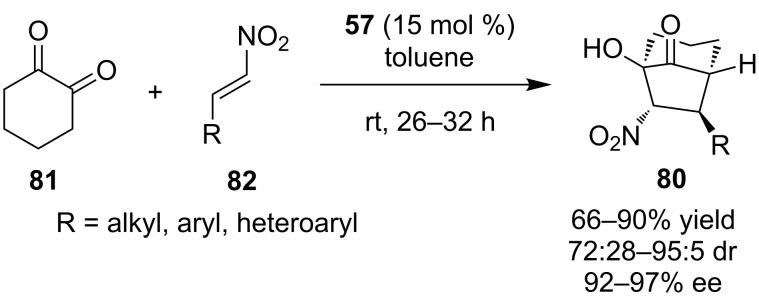
Asymmetric domino Michael–Henry reaction [47].

In 2010, Zhao and his group demonstrated the synthesis of bicyclo[3.2.1]octan-8-ones **80**, via a domino Michael–Henry reaction using quinine-derived catalyst **57** (Scheme 28) [47]. The nucleophile in this process is cyclohexane-1,2-dione (**81**) and the Michael acceptor is nitroolefin **82**. A wide range of substrates were tested and the products were isolated in good yields, moderate diasteroselectivities and excellent enantioselectivities. To expand the utility of the developed process, Zhao and co-workers performed the reaction with *trans*-β-nitrostyrene in gram scale isolating the desired product in 74% yield, 88:12 dr and 96% ee.

**Scheme 29:**
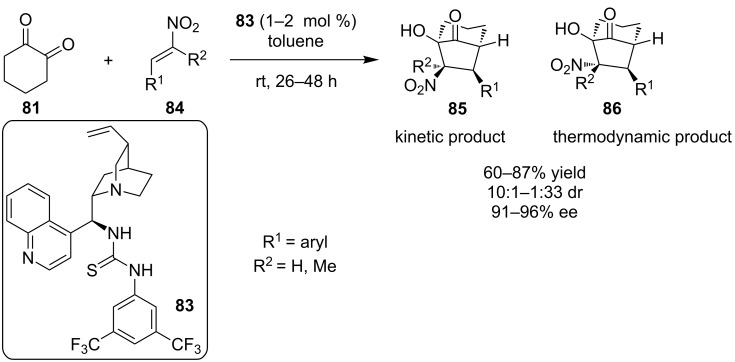
Asymmetric domino Michael–Henry reaction [48].

The same year, Rueping and co-workers utilized the cinchonidine-based thiourea catalyst **83** in much lower catalyst loading, in order to catalyze the same reaction producing the product in high yields and good selectivity (Scheme 29) [48]. In addition, they proposed an explanation for the low diastereoselectivity of the reaction: the kinetic product is slowly interconverting into the thermodynamic product by two pathways: the first one is deprotonation of the α-H to the nitro group and subsequent protonation, and the second pathway is by a retro-Henry process.

**Scheme 30:**
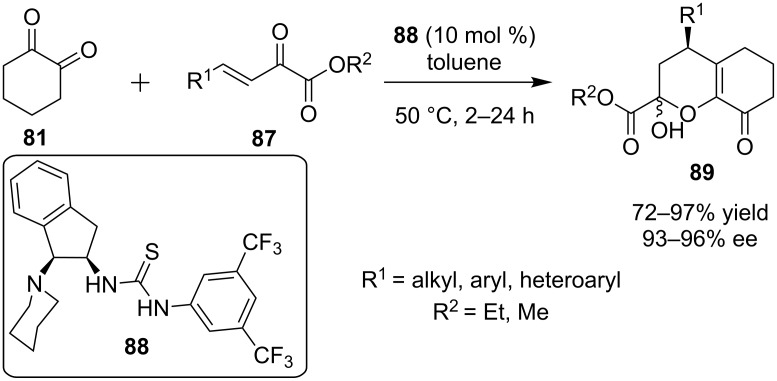
Enantioselective synthesis of derivatives of 3,4-dihydro-2*H*-pyran **89** [49].

Employing the same nucleophile **81**, Wang and his group combined it with β,γ-unsaturated α-ketoesters **87**, as the electrophile, catalyzed by bifunctional indane-derived thiourea **88**, to produce derivatives of 3,4-dihydro-*2H*-pyran **89** (Scheme 30) [49]. This reaction sequence involved a Michael reaction, followed by a hemiacetalization reaction.

The reaction proceeded smoothly for a wide range of substrates to afford the desired products in good to excellent yields (72–97%) and excellent enantioselectivities (93–96% ee). Unfortunately, the product epimerized in the reaction medium, and the resulting product is a mixture of the two anomers.

**Scheme 31:**
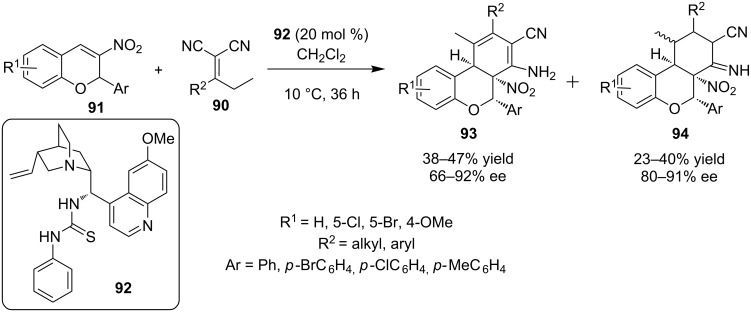
Asymmetric addition of α,α-dicyano olefins **90** to 3-nitro-2*H*-chromenes **91** [50].

In 2012, Xie and his group envisioned the use of α,α-dicyano olefins **90**, as a vinylogous Michael donor in an asymmetric Michael addition to substituted 3-nitro-2*H*-chromenes **91** catalyzed by bifunctional thiourea catalyst **92** (Scheme 31) [50]. When R^2^ is an alkyl group the reaction resulted in the production of **93** and **94** in moderate to excellent enantioselectivities, considering the high molecular complexity achieved in only one step.

**Scheme 32:**
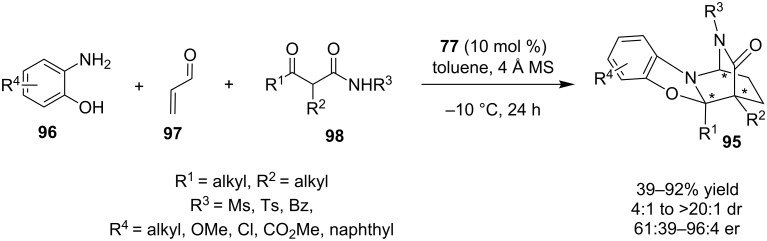
Asymmetric three-component reaction producing 2,6-diazabicyclo[2.2.2]octanones **95** [51].

Recently, Bugaut, Constantieux and co-workers described the enantioselective organocatalytic multicomponent synthesis of 2,6-diazabicyclo[2.2.2]octanones **95**, utilizing Takemoto’s catalyst **77** (Scheme 32) [51]. The reaction was carried out in dry toluene in the presence of molecular sieves at −10 °C, to afford the highly substituted product, containing a 2,6-diazabicyclo[2.2.2] unit and multiple stereocenters, of which two are contiguous and tetrasubstituted, in good yields and selectivities.

**Scheme 33:**
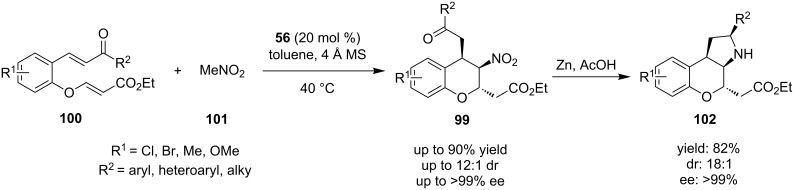
Asymmetric double Michael reaction producing substituted chromans **99** [52].

In 2013, Luo, Xu and co-workers demonstrated an easy method for the synthesis of enantiomerically pure polysubstituted chromans **99**, via the reaction of chalcone enolates **100** and nitromethane (**101**), catalyzed by quinine-derived thiourea **56** (Scheme 33) [52]. Initially nitromethane adds to the chalcone moiety, followed by a nitronate addition to the α,β-unsaturated ester. The substrate scope was widely expanded, including the aromatic moieties containing halogens, alkyl and alkoxy groups. Also, ketones bearing aryl, heteroaryl and alkyl groups, provided the desired products in excellent yields and selectivities. In order to broaden the utility of this methodology, the authors reduced the nitro group to an amine. The product was in situ transformed to the tricyclic product **102**, through a diastereoselective reductive amination, that controlled the stereochemistry of the carbon bearing the R^2^ group.

**Scheme 34:**
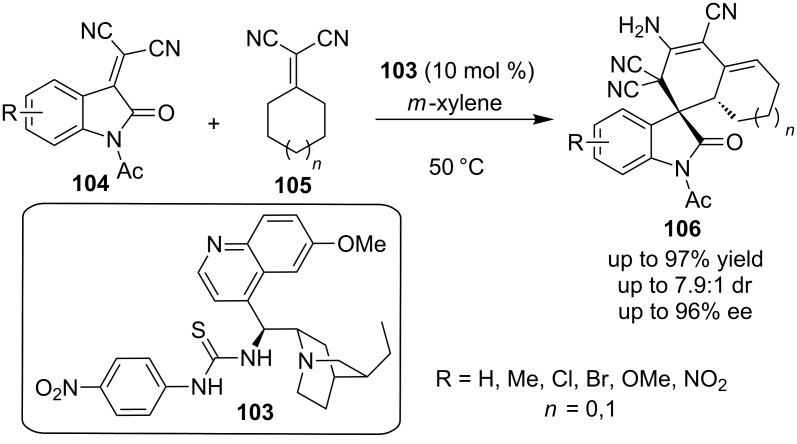
Enantioselective synthesis of multi-functionalized spiro oxindole dienes **106** [53].

Very recently, Wang and co-workers used a cinchona alkaloid-based bifunctional thiourea **103** as the catalyst of choice to an organocatalytic domino process. This domino reaction involded a Michael cyclization–tautomerization reaction sequence between isatylidene malononitriles **104** and α,α-dicyanoalkenes **105**. The process yielded highly functionalized spiro-oxindole dienes **106**. The products were obtained in good to excellent yields (up to 97%) and enantioselectivities (up to 96%), but the diastereoselectivities were moderate (up to 7.9:1) (Scheme 34) [53].

**Scheme 35:**
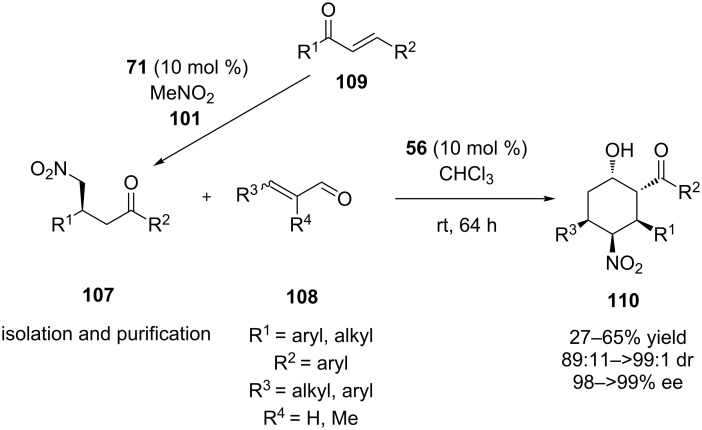
Organocatalyzed Michael aldol cyclization [54].

In 2015, Soós and co-workers disclosed an elegant synthesis of polysubstituted cyclohexanes, utilizing the chiral adduct **107** of the Michael reaction of chalcone **109** catalyzed by a bifunctional thiourea **56** [54]. The authors used a range of different adducts, as well as monosubstituted and disubstituted α,β-unsaturated aldehydes **108**, affording the desired products **110** in moderate to good yields and good to excellent stereoselectivities (Scheme 35).

**Scheme 36:**
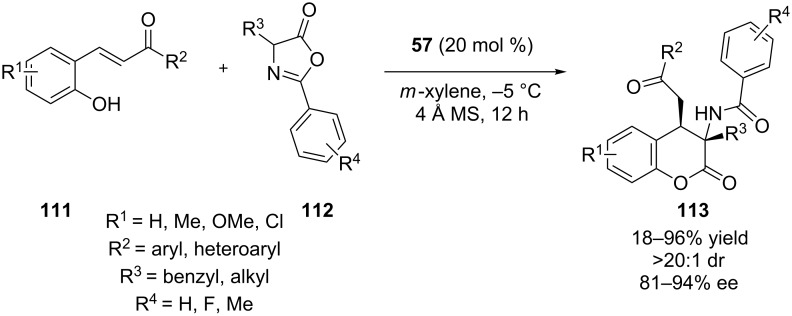
Asymmetric synthesis of dihydrocoumarins [55].

Recently, Wang and co-workers disclosed an asymmetric synthesis of dihydrocoumarins **113** containing adjacent stereogenic centers, utilizing the cinchona-derived bifunctional thiourea **57** [55]. A wide range of azlactones **112** were tested, as well as a plethora of *o*-hydroxychalcone derivatives **111**, providing the products in good to excellent yield and good to excellent stereoselectivity (Scheme 36). The authors proposed that azlactones are deprotonated by the tertiary amine of the organocatalyst to provide an enolate, which in turn reacts with the Michael acceptor **111**.

#### Cascade/domino/tandem reactions producing six-membered rings initiated by Michael addition of activated methylenes and derivatives

**Scheme 37:**
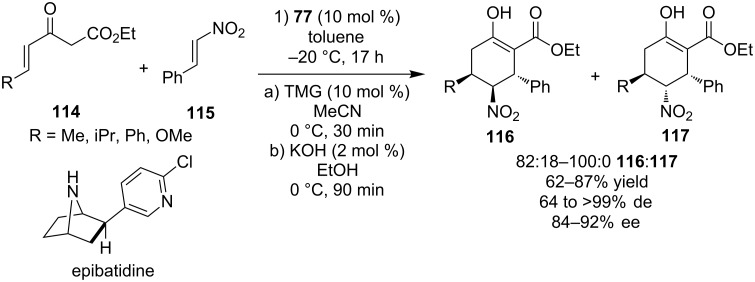
Asymmetric double Michael reaction en route to tetrasubstituted cyclohexenols [56].

In 2004, Takemoto and co-workers demonstrated the enantioselective tandem Michael addition of γ,δ-unsaturated-β-ketoesters **114** to *trans-*β-nitrostyrene **115** which produced tetrasubstituted cyclohexenols **116** and **117** utilizing Takemoto’s catalyst **77** (Scheme 37) [56].

In a paper that described in more detail the transformation, the authors showed that the substitution of the olefin **114** is crucial, in order to proceed the reaction smoothly [57]. The products were isolated in moderate to good yields, excellent diasteroselectivities and good enantioselectivities. With this methodology in hand, the natural product (–)-epibatidine was synthesized.

**Scheme 38:**
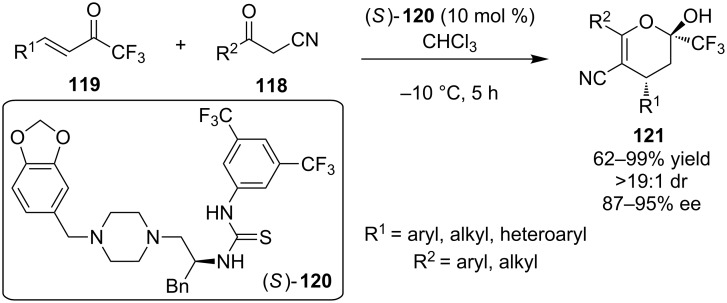
Asymmetric synthesis of α-trifluoromethyl-dihydropyrans **121** [58].

In 2009, Zhao, Zhu and co-workers disclosed the first enantioselective reaction of α-cyanoketones **118** to α,β-unsaturated trifluoromethyl ketones **119**, utilizing a novel organocatalyst that they developed containing a piperazine moiety (*S*)-**120** (Scheme 38) [58]. The reaction proceeded through a Michael addition to the unsaturated ketone, subsequent hemiacetalization and finally elimination to result in the α-trifluoromethyldihydropyrans **121**. The products were isolated in moderate to excellent yields and selectivities.

**Scheme 39:**
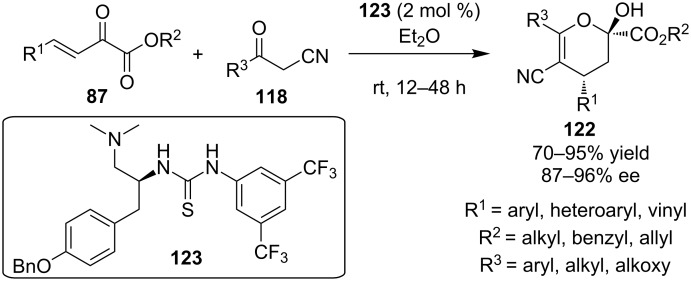
Tyrosine-derived tertiary amino-thiourea **123** catalyzed Michael hemiaketalization reaction [59].

The same year Zhao and co-workers applied the same principles, in order to produce another class of chiral dihydropyrans **122**. They utilized the novel tyrosine-derived tertiary amine-thiourea **123** in quite low catalyst loading to catalyze the reaction between α-cyanoketones **118** and β,γ-unsaturated α-ketoesters **87** (Scheme 39) [59]. Initially a Michael reaction occurs, followed by a hemiacetalization reaction, providing wide range of products in excellent yields (up to 95%) and selectivities (87–96% ee), confirming the generality of the protocol.

**Scheme 40:**
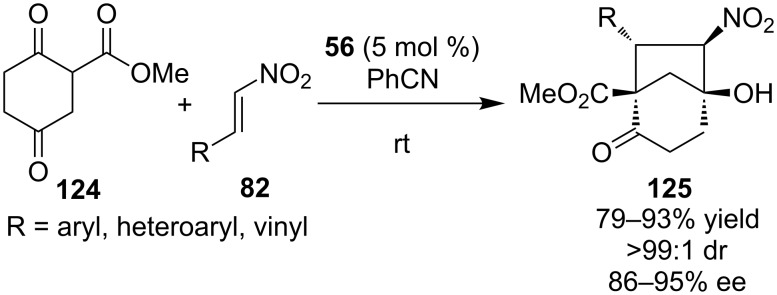
Enantioselective entry to bicyclo[3.2.1]octane unit [60].

In 2010, Zhong and co-workers demonstated that bifunctional thiourea **56** could catalyze the domino Michael–Henry reaction between nitroalkenes **82** and methyl 2,5-dioxocyclohexanecarboxyalate **124** to produce bicyclo[3.2.1]octane unit (Scheme 40) [60]. The reaction proceeded smoothly to afford a wide variety of products **125** in good to excellent yields and selectivities.

**Scheme 41:**
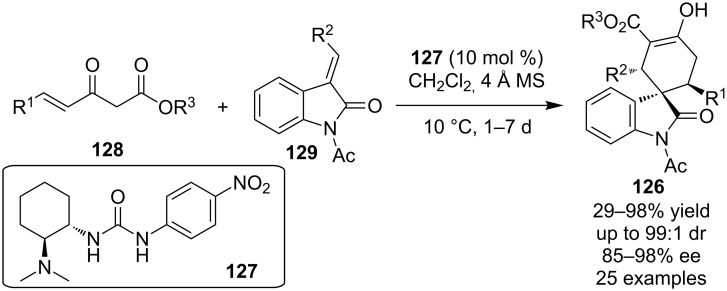
Asymmetric synthesis of spiro[4-cyclohexanone-1,3’-oxindoline] **126** [61].

In 2010, Gong and co-workers developed an asymmetric process en route to spiro[4-cyclohexanone-1,3’-oxindoline] **126** catalyzed by the bifunctional urea **127** (Scheme 41) [61]. The transformation follows a Michael–Michael mechanism and is considered a formal [4 + 2] cycloaddition of **128** (bearing a nucleophilic carbon as well as an electrophilic carbon) and protected methylene-indolinones **129**. A wide range of substrates were tested and the desired products were isolated in good to excellent yields (up to 98%), diastereoselectivities (up to 99:1) and enantioselectivities (up to 98%).

**Scheme 42:**
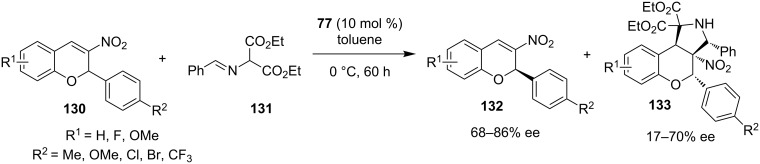
Kinetic resolution of 3-nitro-2*H*-chromene **130** [62].

In 2010, Xie and co-workers reported the kinetic resolution of racemic 3-nitro-2*H*-chromenes **130** catalyzed by Takemoto’s organocatalyst **77** (Scheme 42) [62]. The resulting (*R*)-3-nitro-2*H*-chromene was isolated in rather moderate optical purity.

**Scheme 43:**
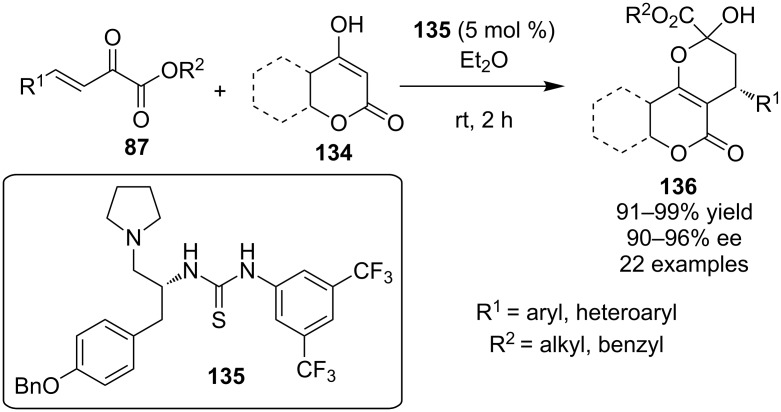
Asymmetric synthesis of chromanes **136** [63].

In 2010, a domino Michael hemiacetalization reaction was reported between cyclic 1,3-dicarbonyl compounds **134** and β-unsaturated α-ketoesters **87** utilizing a novel tyrosine-derived thiourea **135** (Scheme 43) [63].

**Scheme 44:**
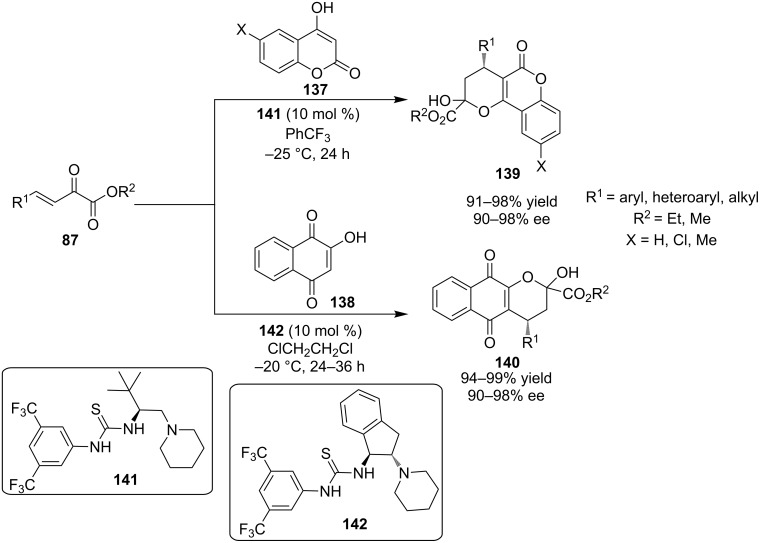
Wang’s utilization of β-unsaturated α-ketoesters **87** [64–65].

In 2010 and 2011, Wang demonstrated that the versatile β-unsaturated α-ketoesters **87** are capable of participating in multiple cascades, initiated by Michael addition of preformed stable enols **137** and **138**. As a result, this methodology provided a highly efficient route to coumarins **139** and napthoquinone derivatives **140** in excellent yields and selectivities (Scheme 44) [64–65]. In both cases, a bifunctional activation of substrates was proposed by the authors.

**Scheme 45:**
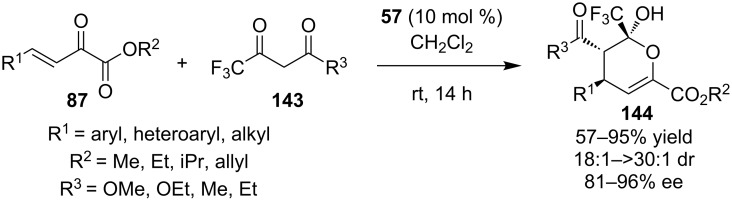
Asymmetric entry to trifluoromethyl-substituted dihydropyrans **144** [66].

In 2011, Yan and co-workers reported the organocatalytic cascade Michael hemiketalization, using the same versatile reagent, β-unsaturated α-ketoester **87**, and 4,4,4-trifluoroacetoacetate **143** to produce trifluoromethyl-substituted dihydropyrans **144** (Scheme 45) [66]. The process is catalyzed by the bifunctional cinchonine-derived thiourea **57**. A number of substrates were presented and the methodology is tolerant to many functional groups.

**Scheme 46:**
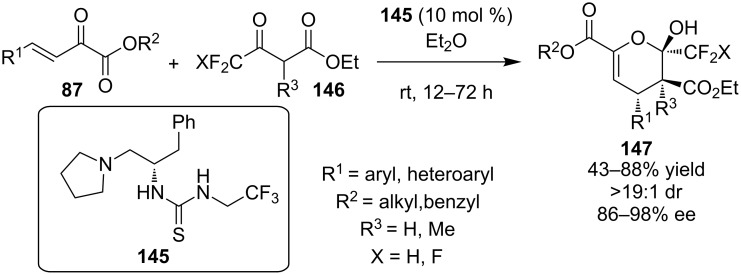
Phenylalanine-derived thiourea-catalyzed domino Michael hemiaketalization reaction [67].

The same year Zhao, Zhu and co-workers developed a new class of thiourea organocatalyst **145** bearing a trifluoromethyl group. The combination of this group and phenylalanine provided an efficient catalyst for the domino reaction between ethyl 4,4,4-trifluoro-3-oxobutanoate **146** and β-unsaturated α-ketoesters **87** (Scheme 46) [67]. A wide range of products were obtained in moderate to good yields and excellent selectivities following this methodology.

**Scheme 47:**
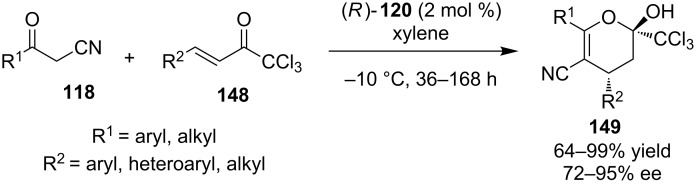
Asymmetric synthesis of α-trichloromethyldihydropyrans **149** [68].

The same year Zhao and co-workers reported a similar type reaction (organocatalytic cascade Michael hemiketalization) between 3-oxo-phenylpropanenitrile **118** and (*E*)-1,1,1-trichloro-4-phenylbut-3-en-2-one **148** catalyzed by bifunctional thiourea (*R)*-**120** producing α-trichloromethyldihydropyrans **149** (Scheme 47) [68]. Utilizing a quite low catalyst loading (2 mol %), good yields and selectivities were achieved.

**Scheme 48:**
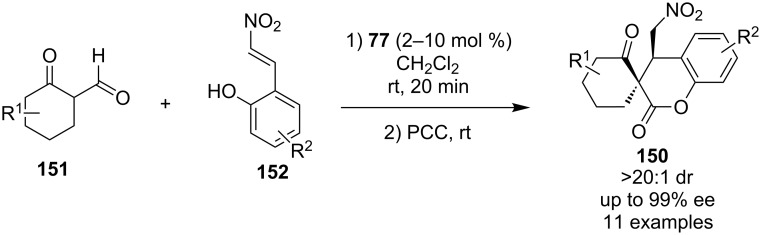
Takemoto’s thiourea-catalyzed domino Michael hemiaketalization reaction [69].

In 2011, Lee and co-workers disclosed the enantioselective synthesis of 3,4-dihydrocoumarins **150** bearing an all-carbon spiro-quaternary stereocenter utilizing Takemoto’s organocatalyst **77** (Scheme 48) [69]. The domino process is initiated by a Michael addition followed by acetalization, and subsequent PCC oxidation in an one-pot transformation.

**Scheme 49:**
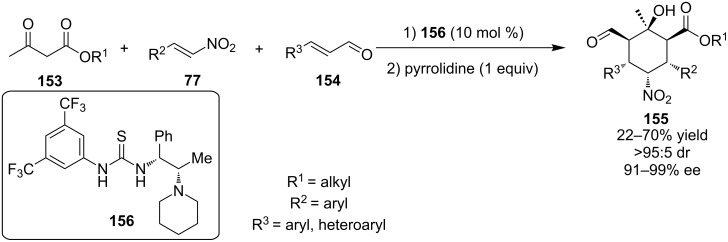
Asymmetric synthesis of densely substituted cyclohexanes [70].

In 2012, Enders and co-workers described the three-component domino Michael–Michael aldol reaction between β-ketoesters **153**, nitroalkenes **77** and α,β-unsaturated aldehydes **154**, producing heavily substituted cyclohexanes **155** containing six contiguous stereocenters with excellent stereocontrol (Scheme 49) [70]. In order to complete the cascade, the authors employed a bifunctional thiourea **156** and pyrrolidine in an one-pot protocol. Overall, the reaction proceeded smoothly and the products were obtained in moderate to good yields (up to 70%), but in excellent selectivities (>95:5 dr and up to 99% ee).

**Scheme 50:**
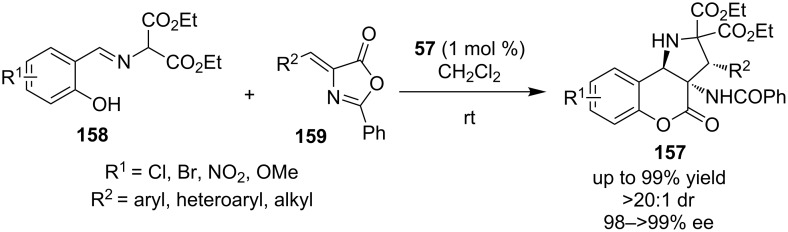
Enantioselective synthesis of polysubstituted chromeno [4,3-*b*]pyrrolidine derivatines **157** [71].

Recently, Liang, Xu and co-workers developed a domino process in order to construct polysubstituted chromeno[4,3-*b*]pyrrolidine derivatives **157**, utilizing a bifunctional organocatalyst **57** (Scheme 50) [71]. The transformation is quite powerful, utilizing under mild conditions and a very low catalyst loading. The transformation is initiated by a Michael addition of **158** to alkylidene azlactone **159**, followed by a Mannich reaction and finally transesterification.

**Scheme 51:**
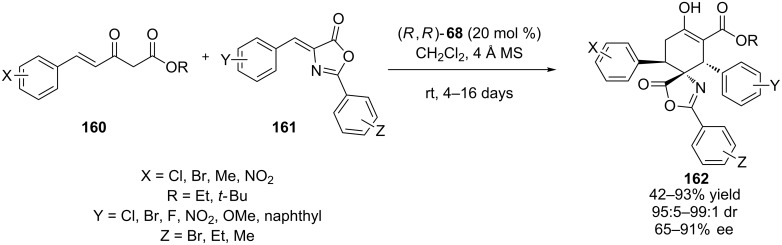
Enantioselective synthesis of spiro-fused cyclohexanone/5-oxazolone scaffolds **162** [72].

The same year, Yuan and co-workers reported the double Michael reaction between **160** and alkylidene azlactone **161** to produce the spiro-fused cyclohexanone/5-oxazolone scaffolds **162** (Scheme 51) [72]. A broad range of both reagents were well tolerated, producing the desired product in moderate to high yields (up to 93%) and diastereoselectivities (up to 99:1 dr) and moderate to good enantioselectivities.

#### Cascade/domino/tandem reactions producing six-membered rings initiated by oxy/aza/sulfa-Michael addition

**Scheme 52:**
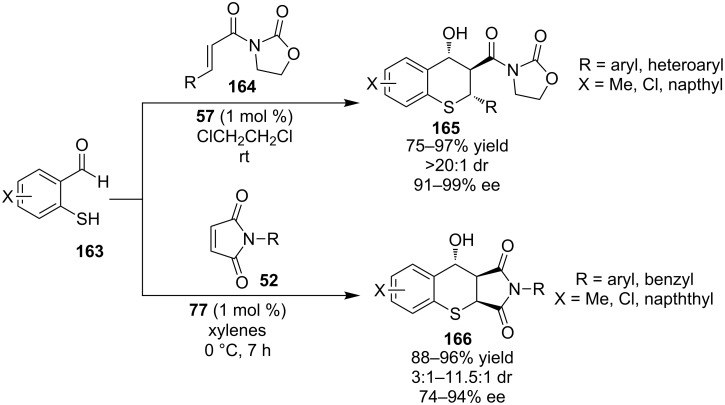
Utilizing 2-mercaptobenzaldehydes **163** in cascade processes [73–74].

In 2007, Wang and co-workers utilized 2-mercaptobenzaldehydes **163** and α,β-unsaturated systems as Michael acceptors, such as α,β-unsaturated oxazolidinones **164** and maleimides **52**, in order to catalyze Michael aldol cascades to construct versatile benzothiopyrans derivatives **165** and **166** (Scheme 52) [73–74]. The reactions operate through a sulfa-Michael aldol mechanism. Those transformations are useful because they produce products containing three contiguous stereocenters in high yields and excellent stereoselectivities utilizing only 1 mol % catalyst loading.

**Scheme 53:**
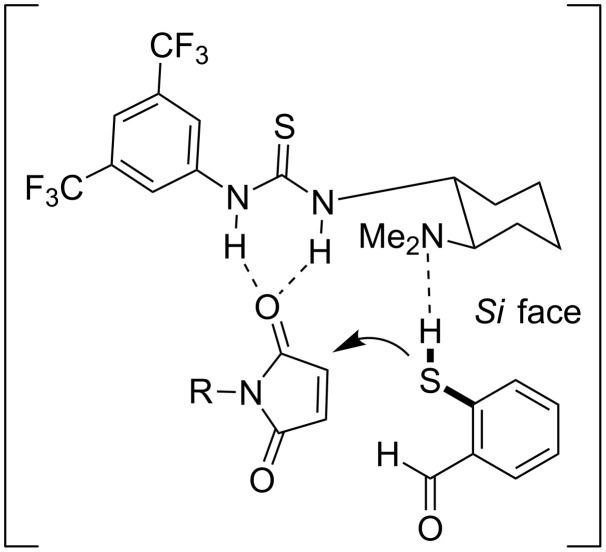
Proposed transition state of the initial sulfa-Michael step [74].

The authors proposed a bifunctional mode of activation. More specifically, the thiourea moiety activates the maleimide through hydrogen-bonding and the tertiary amine recognizes the thiol group, again through hydrogen-bonding, and orients the thiol attacking from the *Si*-face of the maleimides **52** (Scheme 53).

**Scheme 54:**
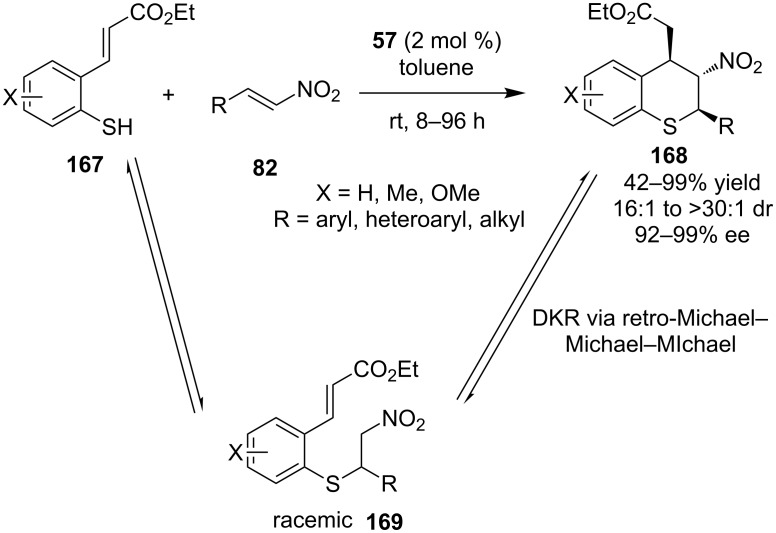
Asymmetric thiochroman synthesis via dynamic kinetic resolution [75].

In 2008, Wang and co-workers described a very interesting Michael–Michael cascade of *trans*-3-(2-mercaptophenyl)-2-propenoic acid ethyl esters **167** and nitroalkenes **82** to produce thiochromane derivatives **168** catalyzed by the bifunctional thiourea **57** (Scheme 54) [75]. The reaction proceeded smoothly for a wide range of substrates with high stereoselectivity, that fact is inconsistent with the current literature as the sulfa-Michael reaction is not catalyzed efficiently by this catalyst. In order to explain the high selectivity of the reaction, they proposed a dynamic kinetic resolution (DKR) pathway of a Michael–*retro-*Michael–Michael–Michael reaction.

**Scheme 55:**
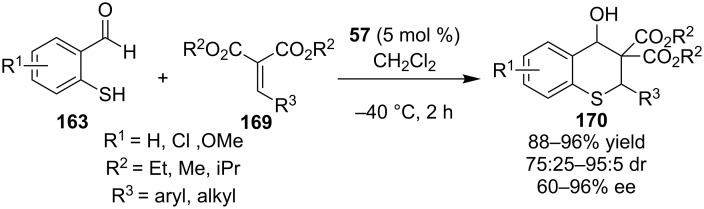
Enantioselective synthesis of thiochromans [76].

The same year Zhao and co-workers reported a novel domino Michael–Knoevenagel reaction between 2-mercaptobenzaldehydes **163** and easily accessible Michael acceptors **169** catalyzed by 9-*epi*-aminoquinine thiourea **57** (Scheme 55) [76]. Various adducts were obtained in good to excellent yields (up to 96%) and moderate to excellent selectivities.

**Scheme 56:**
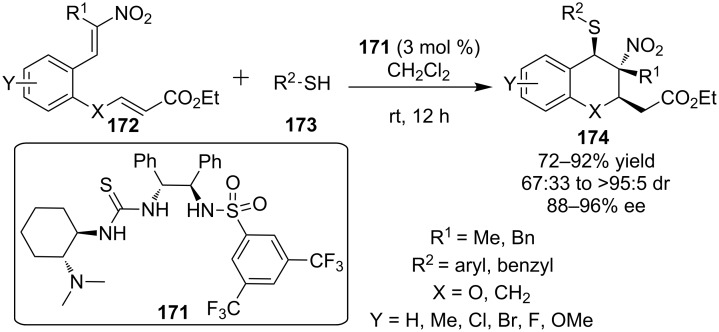
Enantioselective synthesis of chromans and thiochromans synthesis [77].

In 2010, Chen, Xiao and co-workers described a domino sulfa-Michael–Michael reaction catalyzed by the novel multifunctional thiourea **171** (Scheme 56) [77]. The cascade is initiated by the addition of thiol **173** to the more electrophilic double bond of **172**, those in conjugation with the nitro group, and subsequent addition of the nitronate to the remaining double bond.

A wide range of substrates were tested and the desired products **174** were obtained in good to excellent yields (up to 92 %) and selectivities (>95:5 dr and up to 96% ee), employing only 3 mol % catalyst loading. The synthetic utility of the process was further expanded by the multigram version of the reaction utilizing only 0.5 mol % catalyst loading and by the transformations of the adducts into other synthetic intermediates by oxidation either of the nitro group or the thioether group.

**Scheme 57:**
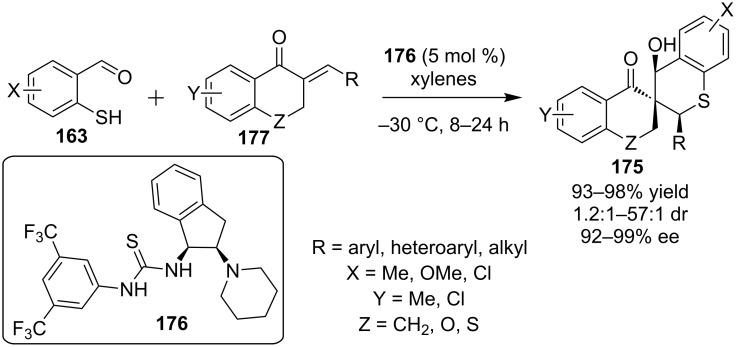
Enantioselective sulfa-Michael aldol reaction en route to spiro compounds [78].

The same year Wang and co-workers reported the enantioselective synthesis of spiro-chromanone-thiochroman compounds **175** catalyzed by a bifunctional indane-based thiourea **176** (Scheme 57) [78]. The cascade is initiated by the sulfa-Michael addition of 2-mercaptobenzaldehyde **163** to the *exo*-α,β-unsaturated ketone **177** and subsequent aldol reaction between the newly-formed enolate and the aldehyde moiety. The desired products were obtained utilizing low catalytic loading (5 mol %) in excellent yields (up to 98%) and enantioselectivities (up to 99% ee), but low to excellent diastereoselectivities (1.2:1–57:1 dr).

**Scheme 58:**
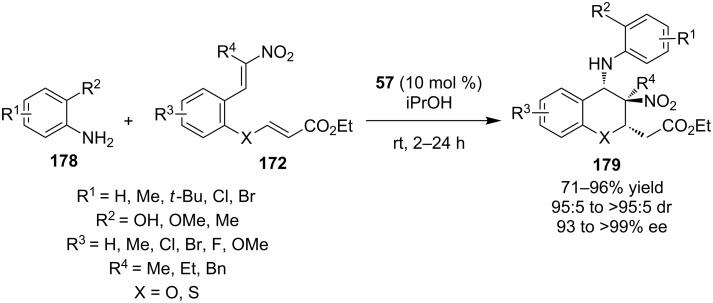
Enantioselective synthesis of 4-aminobenzo(thio)pyrans **179** [79].

In 2011, Chen, Xiao and co-workers, based on their previous work [77], described the aza-Michael–Michael cascade between substituted anilines **178** and nitroolefin enoates **172**, utilizing a bifunctional cinchonine-derived thiourea **57** (Scheme 58) [79]. The reaction proceeds very smoothly for a variety of substrates affording the desired products in excellent yields and selectivities.

**Scheme 59:**
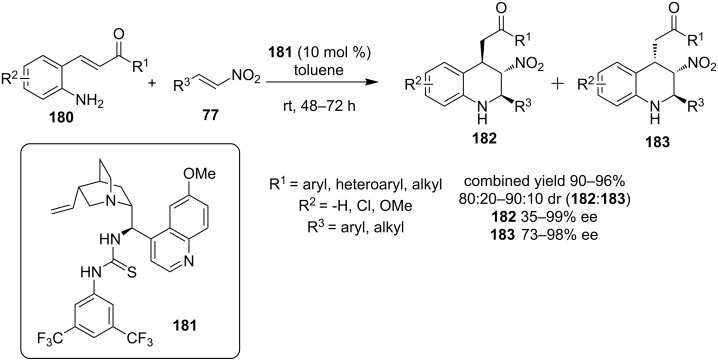
Asymmetric synthesis of tetrahydroquinolines [80].

In 2012, Xu and co-workers described an alternative route to highly-functionalized tetrahydroquinolines employing a domino aza-Michael–Michael reaction of substituted anilines **180** and nitroolefin **77** catalyzed by a bifunctional thiourea **181** (Scheme 59) [80]. The combined yields of the products **182** and **183** was good (up to 96%) but the selectivity was moderate.

#### Miscellaneous cascade/domino/tandem reactions

**Scheme 60:**
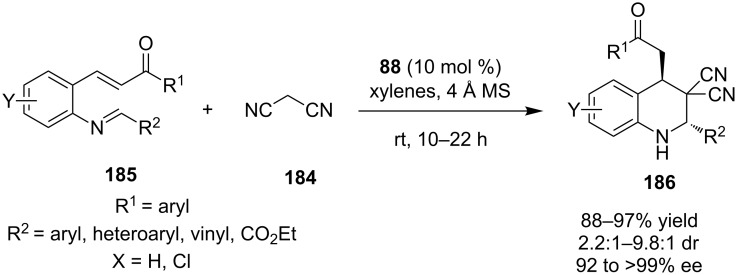
Novel asymmetric Mannich–Michael sequence producing tetrahydroquinolines **186** [81].

In 2012, Wang and co-workers disclosed a novel domino Mannich–Michael reaction between malonitirile **184** and substituted aromatic imine **185** catalyzed by bifunctional thiourea **88** (Scheme 60) [81]. Many functional groups were tolerated, obtaining the desired densely functionalized tetrahydroquinolines **186**. Additional mechanistic studies by the authors strongly suggest the Mannich–Michael pathway instead of the more “reasonable” Michael–Mannich pathway.

**Scheme 61:**
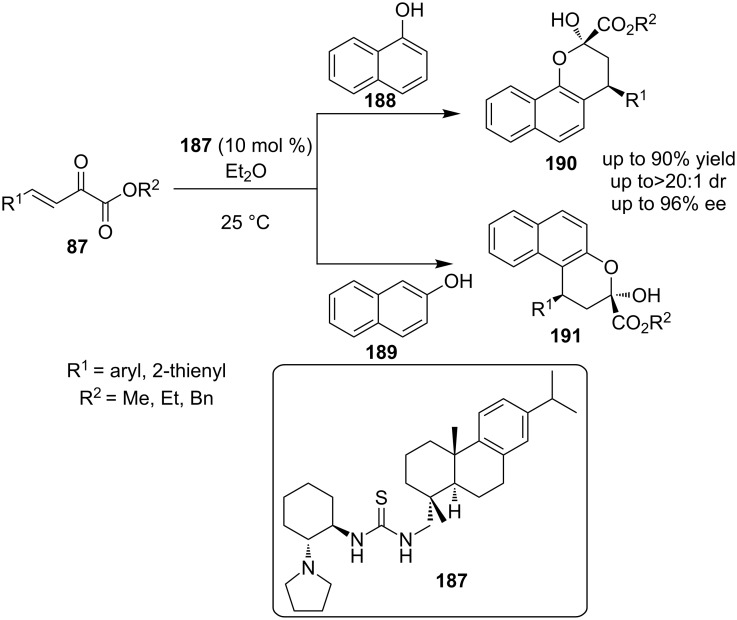
Enantioselective synthesis of biologically interesting chromanes **190** and **191** [82].

In 2012, Wang and co-workers reported a novel domino Friedel–Crafts alkylation (via conjugate addition)-hemiacetalization catalyzed by rosin-derived tertiary amine-thiourea **187** (Scheme 61) [82]. Reagent **87** was successfully combined with nucleophilic naphthols **188** and **189** to produce medicinally interesting chromane derivates **190** and **191** respectively.

**Scheme 62:**
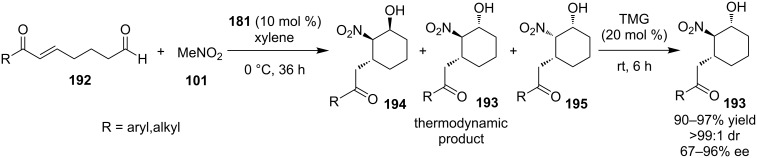
Asymmetric tandem Henry–Michael reaction [83].

Zhao and co-workers employed the bifunctional cinchona-derived thiourea **181** to catalyze the tandem Henry–Michael reaction of nitromethane (**101**) to the enal **192**, but the reaction resulted in three diastereoisomers (Scheme 62) [83]. With this in hand, they envisioned the interconversion of the kinetic products to the most stable product. In order to achieve that, they designed an one-pot two-step process, where upon completion of the tandem Henry–Michael reaction, TMG catalyzed the epimerization to the sole product **193**. Their postulation is based on the fact that Henry reactions are typically reversible, so **194** and **195** could be involved in a retro-Henry and subsequent diastereoselective Henry reaction, where the stereochemical outcome is inducted by the *C*_2_ stereochemistry.

This is further supported by some additional mechanistic experiments they conducted. The substrate scope was also examined and the nature of the R group does not affect the outcome of the reaction, as the reaction proceeds smoothly with excellent selectivity.

**Scheme 63:**
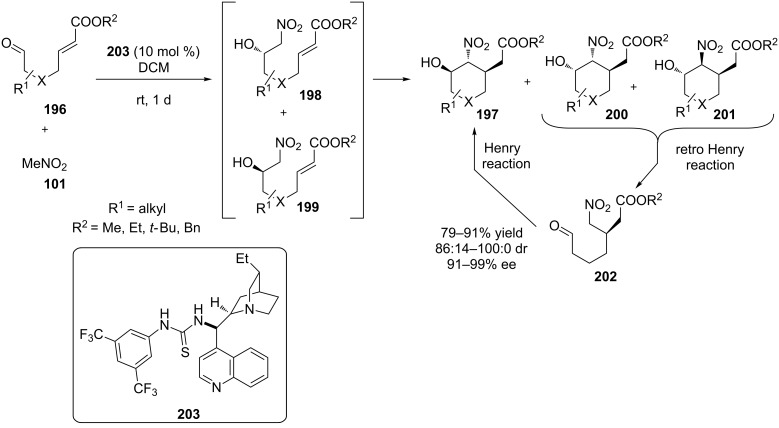
An asymmetric synthesis of substituted cyclohexanes via a dynamic kinetic resolution [84].

In 2013, Quintavalla and co-workers disclosed an interesting Henry–Michael–retro-Henry–Henry domino cascade to furnish substituted cyclohexanes with three adjacent stereocenters [84]. A wide range of aldehydes **196** were tested, obtaining the desired products **197** in good yields and good stereoselectivities (Scheme 63). The process follows an interesting mechanism, proposed by the authors, supported by experimental data. The initial Henry reaction provides the two nitro alcohols **198**, **199** as a mixture of low optical purity. Subsequent Michael addition provides **197**, **200** and **201**. Compounds **200** and **201** equilibrate to **197** via a retro-Henry reaction to **202**, followed by a Henry ring closure.

**Scheme 64:**
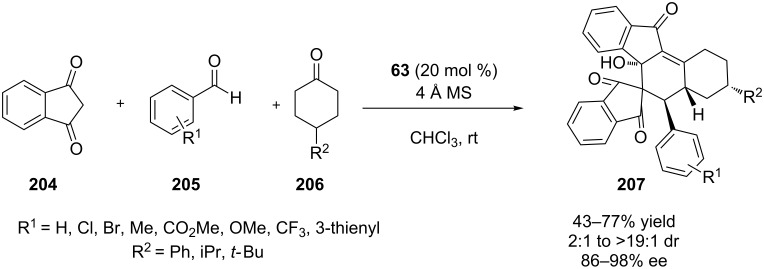
Three component-organocascade initiated by Knoevenagel reaction [85].

In 2015, Chen and co-workers envisaged a three-component organo-cascade quadruple reaction, that yielded highly functionalized polycarbocycles [85]. The authors utilized multiple aromatic aldehydes **205** and some 4-substituted cyclohexanones **206**, affording the desired products **207** in good yield and stereoselectivity, given the high molecular complexity that is being achieved in one step (Scheme 64). The researchers suggested that diketone **204** and benzaldehyde **205** reacts through Knoevenagel condensation, to produce 2-arylidene-1,3-indanediones, which is subsequently attacked by the enolate of cyclohexanone. Two subsequent aldol reactions furnished the desired product.

#### Miscellaneous thiourea-catalysts and catalytic systems promoting asymmetric transformations that lead to a six-membered ring

The discovery of L-proline as an organocatalyst for the aldol reaction was of major importance and therefore many asymmetric reactions that could not be achieved, are now possible. There are many reactions catalyzed by L-proline, affording stereoselective products in high yields and enantiomeric excess, nevertheless there are many limitations. For that reason, it has emerged the need for the synthesis of new molecules that would have the same reactivity with L-proline in catalyzed asymmetric reactions and better properties.

**Scheme 65:**
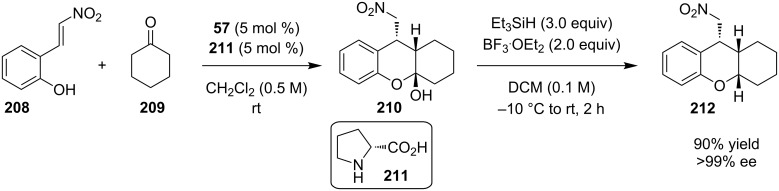
Asymmetric Michael reaction catalyzed by catalysts **57** and **211** [86].

The combination of proline with other molecules to provide a catalytic system was exploited by Ramachary and co-workers in an enamine-based Michael reaction between 2-(2-nitrovinyl)phenol (**208**) and cyclohexanone (**209**, Scheme 65) [86]. When that reaction has been performed under the “regular” conditions for a Michael reaction, product **210** has been obtained in low yields. To overcome this problem, catalysts **57** and **211** were combined and the reaction goes through a more rigid pre-TS assembly. Reduction of the hemiacetal **210**, afforded product **212** in 90% yield and >99% ee.

**Scheme 66:**
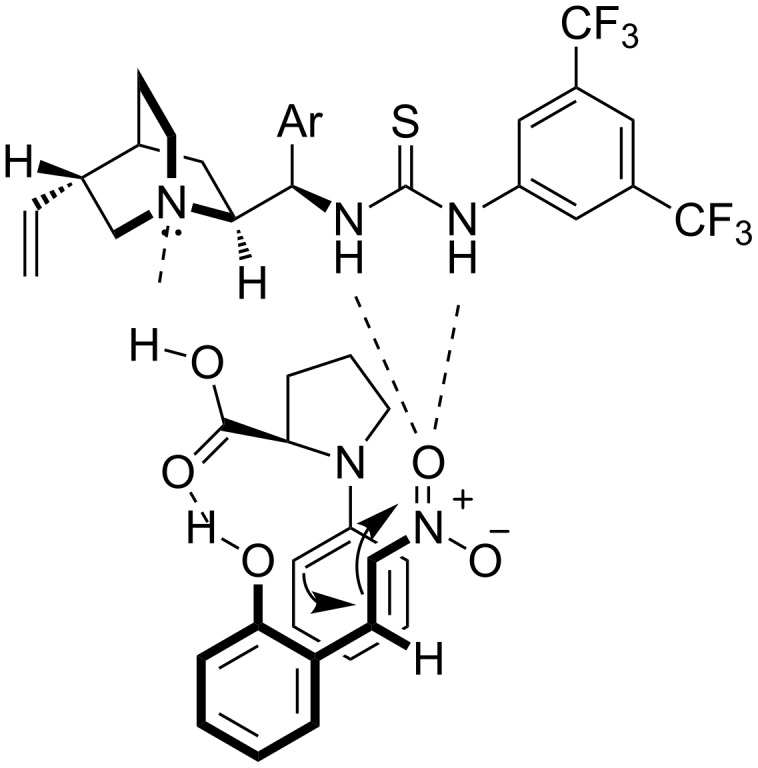
Proposed mechanism for the asymmetric Michael reaction catalyzed by catalysts **57** and **211** [86].

A mechanism for the above reaction, where the *s-cis* enamine attacks the electrophilic double bond of 2-hydroxynitrostyrene, was proposed (Scheme 66).

**Scheme 67:**
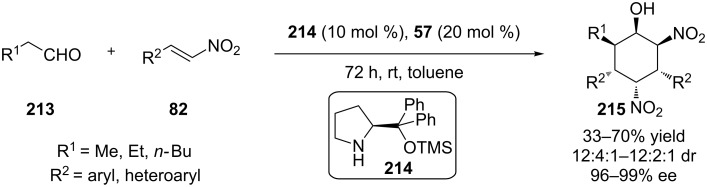
Asymmetric facile synthesis of hexasubstituted cyclohexanes [87].

In 2012, Wang and co-workers developed a dual organocatalyst catalytic system, en route to hexasubstituted hexanes, utilizing some aldehydes **213** and a wide range of nitroolefins **82** [87]. The products were obtained in good yields and good to excellent stereoselectivities (Scheme 67).

**Scheme 68:**
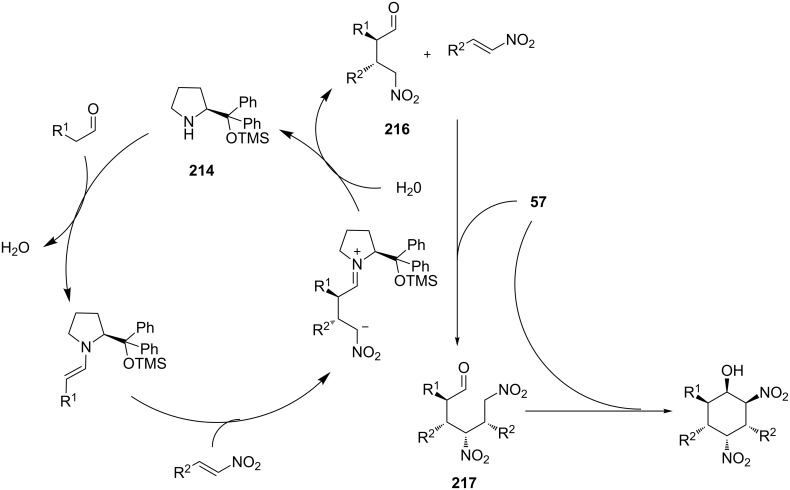
Dual activation catalytic mechanism [87].

The researchers proposed that the diaryl silyl prolinol **214** condenses with the aldehyde to form the corresponding enamine, that in turn reacts with the nitroolefin to produce the Michael adduct **216**. **216** is being deprotonated by the chiral thiourea to afford a nucleophilic nitronate, which attacks the nitroolefin. Subsequent Henry reaction afforded the desired product (Scheme 68).

**Scheme 69:**
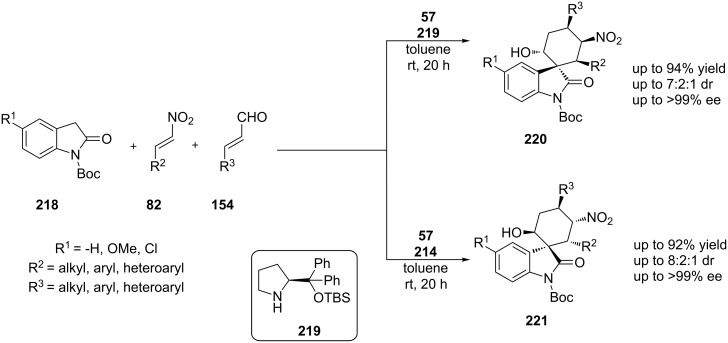
Asymmetric Michael–Michael/aldol reaction catalyzed by catalysts **57**, **219** and **214** [88].

Among the same lines, Zhou, Li and co-workers reported a cascade process affording six-membered spiro-cyclic oxindoles with five adjacent stereocenters. The authors proposed that the reaction proceeds via an asymmetric Michael–Michael aldol sequence (Scheme 69) [88]. In this protocol, when a different derivative of L-diphenylprolinol is used, a different diastereomer of the product is obtained. When along with *N*-Boc-substituted oxindole **218**, substituted derived nitro-alkene **82** and substituted unsaturated aldehyde **154**, a bifunctional quinine-derived thiourea **57** and L-diphenylprolinol-*tert*-butylsilyl ether **219** were used, the substituted *N*-Boc-substituted spiro-oxindoles **220** were obtained. This domino Michael–Michael aldol reaction provides the product in an excellent 94% yield, excellent enantiomeric excess (>99%) and good diastereoselectivity (7:2:1). Utilizing organocatalyst **57** with another derivative of **220**, organocatalyst **214**, another diastereomer was obtained of the desired product **221** (Scheme 69). This domino Michael–Michael aldol reaction provides the product in an excellent 92% yield, excellent enantiomeric excess (>99%) and good diastereoselectivity (9:2.5:1).

**Scheme 70:**
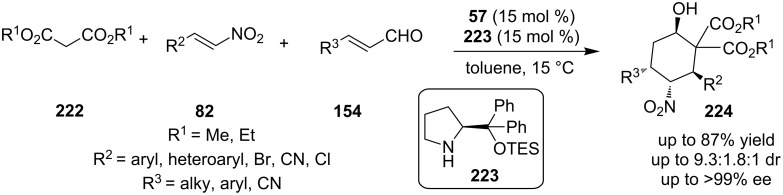
Asymmetric synthesis of substituted cyclohexane derivatives, using catalysts **57** and **223** [89].

Dixon, Xu and co-workers described a three-compound reaction between dialkyl malonate **222**, nitro-alkene **82** and substituted enal **154**, catalyzed by the chiral quinine-derived thiourea **57** and organocatalyst **223**, affording product **224** with a substituted cyclohexane-ring core (Scheme 70) [89]. The experimental results of this reaction were excellent with a 54% yield, 3.1:1:1 dr and >99% ee. The proposed mechanism, begins with an activation of the malonate **222** and the nitro-alkene **82**, so a stereoselective Michael addition occurs. Thus, the formed adduct, through an iminium catalysis pathway caused by catalyst **223**, reacts with the unsaturated aldehyde and affords a pre-aldol substrate. Finally, under basic conditions, an aldol reaction is taking place and gives the final desired substituted cyclohexane.

**Scheme 71:**
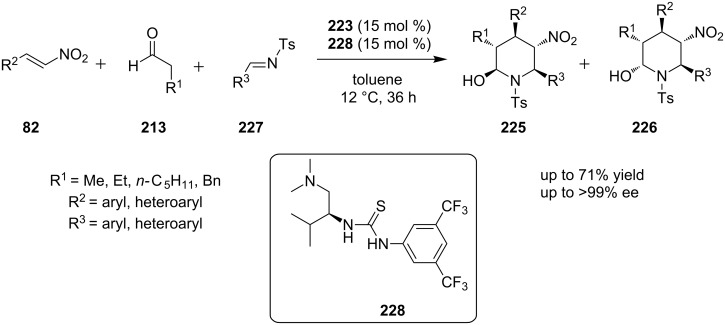
Asymmetric synthesis of substituted piperidine derivatives, using catalysts **223** and **228** [90].

In a similar manner, the same group reported the synthesis of substituted piperidines **225** and **226** through a multiple organocatalytic activation of the substrates which are nitro-alkene **82**, aldehyde **213** and a substituted *(E)*-tosylimine **227** (Scheme 71) [90]. This catalytic reaction gives the product in a good yield and an excellent enantiomeric excess. The proposed mechanism of this reaction starts when catalyst **223** activates aldehyde **213**, through the formation of the corresponding enamine. Then, the enamine reacts with nitro-alkene **82**, which is activated by hydrogen bonding due to catalyst **228**. Thus, the formed intermediate can now participate to a nitro-Mannich reaction, affording a *N*-protected aminoaldehyde product. Finally, the *N*-protected aminoaldehyde product can now be cyclized under the reactions’ conditions.

**Scheme 72:**
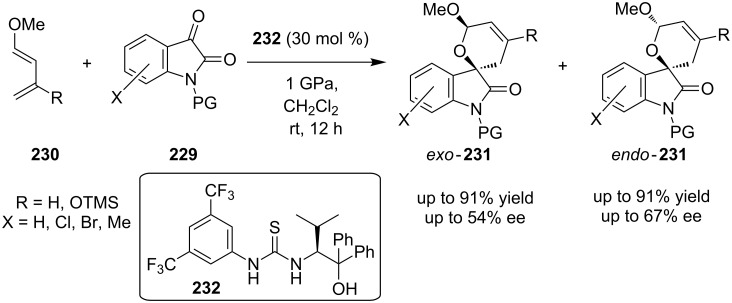
Asymmetric synthesis of *endo*-*exo* spiro-dihydropyran-oxindole derivatives catalyzed by catalyst **232** [91].

Another stereoselective reaction was attempted by Kotsuki’s group presenting an organocatalytic hetero-Diels–Alder reaction between isatin **229** with substituted diene **230**. High pressure had to be employed in order to obtain spiro-dihydropyran-oxindole derivatives **231** in good to excellent yields, using catalyst **232** (Scheme 72) [91]. The mechanistic studies showed that the 3,5-bis(trifluoromethyl)phenyl group was an essential component of the thiourea catalyst. After the optimization of the reaction conditions the yields of products **231** were 71–91%.

**Scheme 73:**
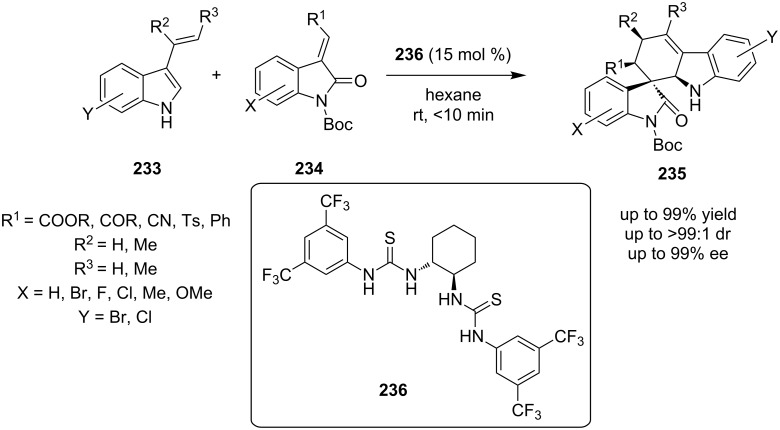
Asymmetric synthesis of carbazole spiroxindole derivatives, using catalyst **236** [92].

Barbas and co-workers reported the synthesis of carbazole spiro-oxindole derivatives, in a Diels–Alder reaction in very short reaction time (10 min). The reagents were the substituted indoles **233,** benzylidene oxindolinones **234** and the organocatalyst, a *C*_2_-symmetric bis-thiourea **235** was employed to yield product **236** (Scheme 73) [92]. Suprisingly, a single diastereoisomer was isolated, despite the fact that four new chiral centers were produced. The products were obtained in high yields (75–99%) and ee values 88–99%. The biggest advantage of this reaction is that it can be transferred to large-scale chemical production, due to the difference in the solubilities of the reactants and the products, which means the product and the catalyst can be isolated separately.

**Scheme 74:**
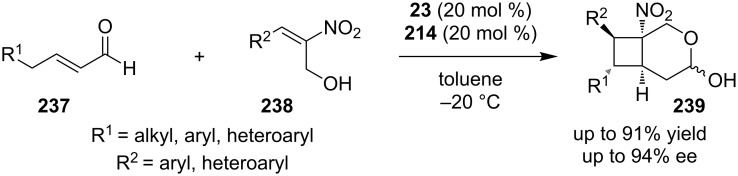
Enantioselective formal [2 + 2] cycloaddition of enal **209** with nitroalkene **210**, using catalysts **23** and **214** [93].

In 2012, Carrillo and co-workers reported an enantioselective formal [2 + 2] cycloaddition of enals **237** with nitroalkenes **238** to obtain the oxabicyclo product **239** (Scheme 74) [93]. A combination of catalysts was used, with catalysts **23** and **214**. This reaction affords the desired product in 38–91% yield and 85–95% ee.

**Scheme 75:**
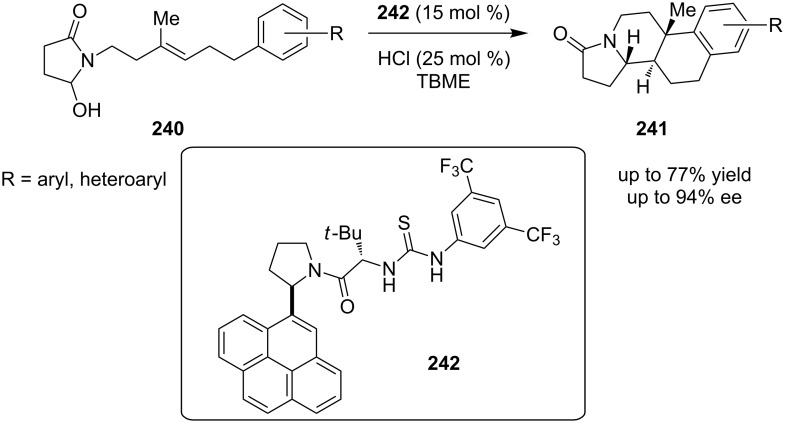
Asymmetric synthesis of polycyclized hydroxylactams derivatives, using catalyst **242** [94].

Furthermore, a thiourea catalyzed reaction via a cationic polycyclization of hydroxylactams **240** leads to the corresponding polycyclized products **241**, using organocatalyst **242** (Scheme 75) [94]. The authors postulated that the existence of an extended aromatic framework on the catalyst is very crucial, as the delocalized π-electron system interacts with the *N*-acyliminium ion intermediate through a stabilizing cation–π-interaction. They came to this conclusion, after an extensive catalyst screening.

**Scheme 76:**
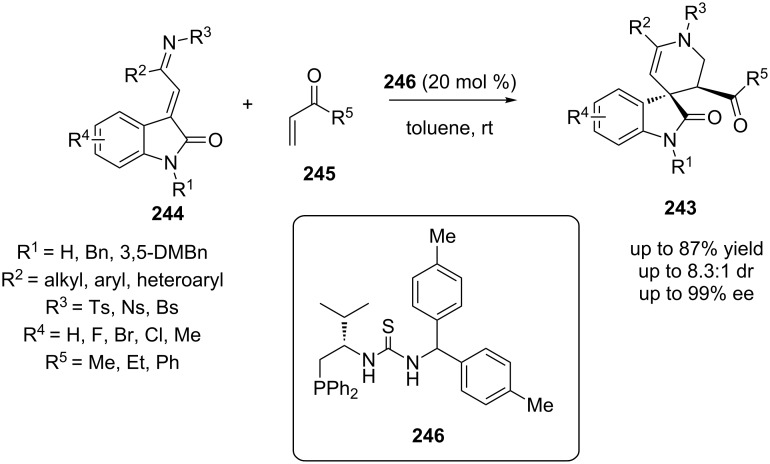
Asymmetric synthesis of product **243**, using catalyst **246** [95].

In 2014, Shi and co-workers presented the synthesis of products **243**, utilizing substrates **244** and α,β-unsaturated aldehyde **245**. Chiral phosphine organocatalyst **246** was employed as the catalyst (Scheme 76) [95]. Product **243** was obtained in high yield (85%), high ee values (up to 99%) and high diastereoselectivity (8.1:1).

**Scheme 77:**
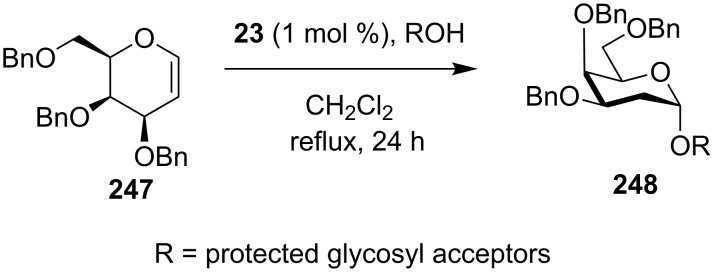
Formation of the α-stereoselective acetals **248** from the corresponding enol ether **247**, using catalyst **23** [96].

In 2012, an interesting α-selective approach for the synthesis of galactopyranoses using achiral thiourea organocatalyst **20**, was reported from McGarrigle, Galan and co-workers (Scheme 77) [96]. In this reaction the reagent is 2,3,4-trisubstituted dihydro-pyran **247** and the product is the corresponding α-galactopyranose **248**. This reaction provides exclusively the α-diastereomer in a yield up to 98%.

**Scheme 78:**
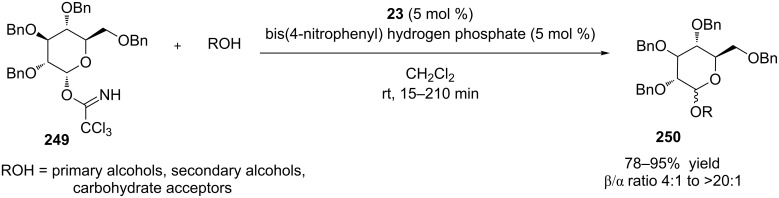
Selective glycosidation, catalyzed by Shreiner’s catalyst **23** [97].

In 2013, Schmidt and co-workers described the use of Shreiner’s thiourea as a catalyst in glycosidation with *O*-glycosyl trichloroacetamides as glycosyl donors [97]. α-D-glucopyranosyl trichloroacetimidate **249** was employed as a donor, several alcohols were utilized, achieving moderate to excellent anomeric selectivity (Scheme 78). Other *O*-glycosyl donors were tested, giving similar results.

## Conclusion

Throughout this review, efficient ways of activating both substrates by interactions via hydrogen bonds, derived from thiourea moieties, were presented. Reactions providing enantiopure products were shown to be catalyzed by primary, secondary and tertiary chiral amine-thioureas, or a combination of catalysts. Products were obtained in one-pot or step-economic domino processes, achieving high increase of molecular complexity in step-economy transformations. There is no doubt that this scientific field will grow in the near future, providing more efficient ways of constructing six-membered rings.
